# Uncovering the Molecular Mechanism of the Qiang-Xin 1 Formula on Sepsis-Induced Cardiac Dysfunction Based on Systems Pharmacology

**DOI:** 10.1155/2020/3815185

**Published:** 2020-08-27

**Authors:** Shasha He, Jingxia Zhao, Xiaolong Xu, Xuran Cui, Ning Wang, Xuyang Han, Yuhong Guo, Qingquan Liu

**Affiliations:** ^1^Beijing Hospital of Traditional Chinese Medicine, Capital Medical University, Beijing, China; ^2^Beijing Institute of Traditional Chinese Medicine, Beijing, China; ^3^Beijing Key Laboratory of Basic Research with Traditional Chinese Medicine on Infectious Diseases, Beijing, China

## Abstract

Cardiac dysfunction is a critical manifestation of sepsis-induced multiorgan failure and results in the high mortality of sepsis. Our previous study demonstrated that a traditional Chinese medicine formula, Qiang-Xin 1 (QX1), ameliorates cardiac tissue damage in septic mice; however, the underlying pharmacology mechanism remains to be elucidated. The present study was aimed at clarifying the protective mechanism of the QX1 formula on sepsis-induced cardiac dysfunction. The moderate sepsis model of mice was established by cecal ligation and puncture surgery. Treatment with the QX1 formula improved the 7-day survival outcome, attenuated cardiac dysfunction, and ameliorated the disruption of myocardial structure in septic mice. Subsequent systems pharmacology analysis found that 63 bioactive compounds and the related 79 candidate target proteins were screened from the QX1 formula. The network analysis showed that the QX1 active components quercetin, formononetin, kaempferol, taxifolin, cryptotanshinone, and tanshinone IIA had a good binding activity with screened targets. The integrating pathway analysis indicated the calcium, PI3K/AKT, MAPK, and Toll-like receptor signaling pathways may be involved in the protective effect of the QX1 formula on sepsis-induced cardiac dysfunction. Further, experimental validation showed that the QX1 formula inhibited the activity of calcium/calmodulin-dependent protein kinase II (CaMKII), MAPK (P38, ERK1/2, and JNK), and TLR4/NF-*κ*B signaling pathways but promoted the activation of the PI3K/AKT pathway. A cytokine array found that the QX1 formula attenuated sepsis-induced upregulated levels of serum IFN-*γ*, IL-1*β*, IL-3, IL-6, IL-17, IL-4, IL-10, and TNF-*α*. Our data suggested that QX1 may represent a novel therapeutic strategy for sepsis by suppressing the activity of calcium, MAPK, and TLR4/NF-*κ*B pathways, but promoting the activation of AKT, thus controlling cytokine storm and regulating immune balance. The present study demonstrated the multicomponent, multitarget, and multipathway characteristics of the QX1 formula and provided a novel understanding of the QX1 formula in the clinical application on cardiac dysfunction-related diseases.

## 1. Introduction

Sepsis, defined as life-threatening organ dysfunction caused by a dysregulated host response to infection, affects more than 19 million people per year and is the main cause of death in intensive care units [1, 2]. Cardiac dysfunction is critical to sepsis-induced multiorgan failure. Cardiac dysfunction occurs in over 40% of sepsis patients, which is associated with high mortality and poor prognosis [3]. Despite improvements in antibiotic therapies and critical care techniques, the management of cardiac dysfunction in patients with sepsis remains challenging since basic interventions for cardiac dysfunction or sepsis alone are contradictory in key areas, including fluid resuscitation [4]. The pathological mechanisms of cardiac dysfunction in sepsis are multifactorial, including inflammatory mediator disorder, mitochondrial dysfunction, apoptosis, and calcium regulation disorder [5, 6]. Therefore, developing a drug that can inhibit these pathological changes would be of great clinical significance for the prevention of sepsis-induced cardiac dysfunction.

Traditional Chinese medicine (TCM) is an integral medicine system with clinical practice over thousands of years. Our previous study showed that the TCM prescription Qiang-Xin 1 (QX1) ameliorates cardiac tissue damage in mice suffering from sepsis partly by inhibiting endoplasmic reticulum- and mitochondria-related apoptosis [7]. However, a holistic understanding underlying mechanisms of the QX1 formula in improving sepsis-induced cardiac dysfunction still is needed in further study.

Systems pharmacology, an emerging systematic methodology combining pharmacology and systems biology, provides a holistic analysis approach to explore the molecular mechanism of TCM [8]. Systems pharmacology includes pharmacokinetics evaluation (absorption, distribution, metabolism, excretion, and toxicity [ADME/T] characteristics of herbs), target protein prediction, and network analysis. At present, systems pharmacology has been widely used to reveal the potential mechanism of TCM formulas in the treatment of cancer, inflammatory bowel disease, and cardiovascular disease [9–11].

**Figure 1:**
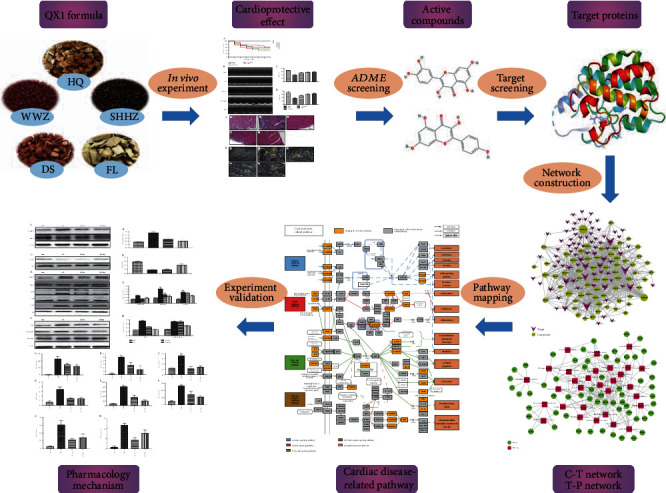
Workflow of the current study.

The present study was aimed at investigating the molecular mechanism of the QX1 formula in the treatment of sepsis-induced cardiac dysfunction. First, the effect of the QX1 formula on survival rate and cardiac dysfunction was assessed in septic mice. Then, the material basis and potential interaction mechanism of the QX1 formula were analyzed by systems pharmacology. Finally, we further verified the mechanism of the QX1 formula on the main signaling pathways integrated by systemic pharmacology in septic mice. The workflow of the current study was shown in Figure 1.

## 2. Materials and Methods

### 2.1. Animals and Ethics Statement

BALB/c mice (male, 18–22 g, 8 weeks old) provided by Beijing HFK Bioscience Co., Ltd. (Beijing, China) were housed under a pathogen-free environment with free access to food and water. All procedures performed on the animals were conducted in accordance with the National Institutes of Health Guidelines on Laboratory Research and approved by the Animal Care and Use Committee of the Beijing Institute of Traditional Chinese Medicine (permit number: 2018040206).

### 2.2. Preparation of the QX1 Formula

The QX1 formula is composed of five herbs: *Astragalus membranaceus* (Fisch.) (HQ), *Polygonum orientale* L. (SHHZ), *Poria cocos* (Schw.) Wolf (FL), *Salvia miltiorrhiza* Bge. (DS), and *Schisandra chinensis* (Turcz.) Baill. (WWZ). All herbs were obtained from the Chinese Pharmacy of Beijing Hospital of Traditional Chinese Medicine and were mixed in the proportion of 3 : 3 : 2 : 2 : 1, with a total weight of 110 g. After soaking for 1 h, the QX1 decoction was prepared by water extraction twice. The extract was then filtered and condensed to 110 ml, with a concentration equal to 1 g herb/ml.

### 2.3. Cecal Ligation and Puncture- (CLP-) Induced Sepsis

A mouse model with moderate sepsis was established by cecal ligation and puncture (CLP) surgery according to the protocol described previously [12]. Briefly, mice were anesthetized with 1% pentobarbital sodium, and a 1–2 cm longitudinal skin midline incision was made to expose the internal organs. The cecum was exposed and ligated in the mid position, which comprised 50% of the cecum, and punctured through and through with a 21-gauge needle. Then, a small amount of feces was extruded from the puncture holes to make sure patency. The cecum was transferred to the abdominal cavity, and the peritoneum and skin were closed by applying sutures. After surgery, mice were injected with sterile saline solution (0.9%, 24 ml/kg of body weight) for fluid resuscitation. In the sham group, the procedure was carried out in the same way as the CLP described above, except without ligation and puncture of the cecum.

### 2.4. Treatment Protocol of the QX1 Formula

After a 7-day acclimation period, 90 mice were randomly assigned to five groups: (1) sham group (sham, *n* = 10), wherein mice received a sham operation without drug treatment; (2) CLP group (CLP, *n* = 20), wherein mice received a CLP operation without QX1 decoction treatment; (3) low-dose QX1 decoction group (QX1 Low, *n* = 20), wherein mice received a CLP operation with 5 g/kg QX1 decoction treatment; (4) high-dose QX1 decoction group (QX1 High, *n* = 20), wherein mice received a CLP operation with 10 g/kg QX1 decoction treatment; and (5) trimetazidine group (TMZ, *n* = 20), wherein mice received a CLP operation with 20 mg/kg TMZ treatment. The mice in QX1 Low, QX1 High, and TMZ groups were orally administered intragastrically with different concentrations of QX1 decoction at 6 and 18 h after the CLP operation, respectively, whereas mice in the sham and CLP groups were administered with the same volume of water. In a survival test, another 20 mice from each group were used to assess survival rates during seven days.

### 2.5. Sample Collection

At 24 h after the CLP operation, mice were anesthetized with 1% pentobarbital sodium and blood samples were collected. Serum was separated for quantitative analysis of cytokines. The heart tissues were harvested and divided into three parts: one was stored in 10% buffered formalin phosphate for histological analysis, one was fixed in 4% glutaraldehyde for ultrastructure analysis, and the other was stored at −80°C for Western blot analysis.

### 2.6. Hematoxylin and Eosin (H&E) Staining

The heart samples were immersed in 10% neutral buffered formaldehyde at room temperature for 48 h, and the fixed samples were then embedded in liquid paraffin and sectioned into 5 *μ*m thickness. The sections were stained with hematoxylin and eosin, and the cardiac morphological changes were observed under a light microscope (Zeiss GmbH, Jena, Germany).

### 2.7. Transmission Electron Microscopy (TEM)

The cardiac tissue samples were fixed with 4% glutaraldehyde overnight, postfixed in cold 1% osmium tetroxide, and then washed with cacodylate buffer three times. Subsequently, cardiac tissue was dehydrated in a series of graded acetone and embedded in an epoxy resin. Ultrathin sections were stained with saturated uranyl acetate in 50% ethanol and lead citrate and observed under an HT7700 transmission electron microscope (Hitachi, Tokyo, Japan).

### 2.8. Echocardiography Analysis

At 24 h after CLP or sham surgery, echocardiography was performed using the Vevo 770 ultrasound system (Visual Sonics Inc., Toronto, Canada) to assess the cardiac function. Briefly, mice were anesthetized with isoflurane at a concentration of 4% (induction) or 1.5% (maintenance) in 100% oxygen. The left ventricular (LV) M-mode tracing was gained from the transthoracic parasternal short-axis view. Through these images, the left ventricular internal dimensions at diastole/systole (LVIDd/LVIDs) and the left ventricular volume at diastole/systole (LVVd/LVVs) were measured and used to determine the left ventricular ejection fraction (LVEF) and left ventricular fractional shortening (LVFS). Each parameter was recorded in least three consecutive cardiac cycles.

### 2.9. Database Construction

The chemical ingredients of all herbs in the QX1 formula were data-mined from the Traditional Chinese Medicine Systems Pharmacology Database (TCMSP, http://lsp.nwu.edu.cn/tcmspsearch.php) and a large number of related literature mining, including PubMed and China National Knowledge Infrastructure (CNKI) databases. Finally, we obtained 513 chemical ingredients and their physicochemical properties from QX1: 87 compounds of HQ, 130 compounds of WWZ, 202 compounds of DS, 34 compounds of FL, and 60 compounds of SHHZ.

### 2.10. Active Compound Screening

#### 2.10.1. Oral Bioavailability (OB)

OB is one of the most important pharmacokinetic parameters in ADME (absorption, distribution, metabolism, and excretion) characteristics, which indicates the efficiency of active drug delivery to the systemic circulation. In the present study, the OBioavail1.1 model was used to estimate OB values [13]. And compounds from QX1 satisfy OB ≥ 28% as a candidate active molecule for subsequent step screening.

#### 2.10.2. Druglikeness (DL)

DL is used to assess the similarity of physical properties of compounds with known drugs. According to previous reports, the drug-like active molecules were picked out from QX1 based on molecular descriptors and the Tanimoto coefficient [14]. In this study, a compound with DL ≥ 0.18 was selected as the active compound of herbs for further study.

#### 2.10.3. Drug Half-Life

Half-life refers to the time it takes for the concentration of a drug to be degraded to half in the body and is considered to be an essential pharmaceutical property, which is mainly used as a time measure for defining the efficacy of a compound. The HL ≥ 4 was adopted as the criterion to screen the candidate active compound of QX1 in this study.

#### 2.10.4. Caco-2 Cell Permeability

The human intestinal cell line Caco-2 is commonly used as an effective in vitro model to study the passive diffusion of drugs through the intestinal epithelium. We used the transport rate of drug molecules in Caco-2 cell monolayers as an evaluation of intestinal absorption. Those chemical ingredients with Caco-2 cell permeability ≥ –0.4 were filtered out as candidate active compounds.

### 2.11. Target Prediction

To identify the target molecules of the candidate active compounds is a key step to reveal the mechanism of QX1. Currently, the weighted ensemble similarity (WES) model was applied to predict the potential targets of QX1 compound [15]. Then, a similarity based on chemical fingerprinting is used to obtain potential targets (http://sea.bkslab.org/search/). Finally, the targets from different sources were named uniformly in the UniProt database (http://www.uniprot.org) and then submitted to the Pharmacogenomics Knowledgebase (PharmGKB, https://www.pharmgkb.org/), Therapeutic Targets Database (TTD, http://database.idrb.cqu.edu.cn/TTD/), and Comparative Toxicogenomics Database (CTD, http://ctdbase.org/) to remove redundant and erroneous targets, so as to ensure the accuracy of the target database.

### 2.12. Network Construction

Traditional Chinese medicine (TCM) is a whole system with multicompound and multitarget characteristics. There is a complicated relationship between effective active compounds, active targets, and pathways. Therefore, the network visualization analysis software Cytoscape was used to draw the compound-target (C-T) network and target-pathway (T-P) network.

In order to investigate the molecular mechanism of the QX1 formula against cardiac injury, an integrated “cardiac disease-related pathway” was established. Firstly, the active targets were mapped to the KEGG database (http://www.kegg.jp/). Then, according to the latest pathological information of a cardiac disease-related pathway, an integrated compound-target pathway diagram was constructed by combining C-T network and T-P network analyses.

### 2.13. Target-Tissue Location

To understand QX1 formula therapy for cardiac disease at the organ level, first, GO analysis showed the most obvious targets among the screened compound targets, and then, their distribution in tissues and organs was analyzed. The tissue distribution of the targets was identified based on microarray analysis of different tissue types in the BioGPS database (http://biogps.org).

### 2.14. Ultraperformance Liquid Chromatography Coupled with Orbitrap Q Exactive Mass Spectrometry (UPLC-MS)

Plasma samples were collected at 0, 15, 30, 60, and 120 min after oral administration with 10 g/kg QX1 decoction. The reference standards of quercetin, formononetin, kaempferol, taxifolin, cryptotanshinone, and tanshinone IIA were purchased from the National Institutes for Food and Drug Control (Beijing, China). The plasma samples and standard solutions were analyzed using ultraperformance liquid chromatography coupled with Orbitrap Q Exactive mass spectrometry (Thermo Scientific, San Jose, USA). Briefly, acetonitrile (A) and 0.1% formic acid aqueous solution (B) were selected as the mobile phases. The gradient mobile phase was as follows: 0% A from 0 to 1 min, 0% to 95% A from 1 to 10 min, 95% to 98% A from 10 to 14.5 min, 98% to 0% A from 14.5 to 14.6 min, and 0% A from 14.6 to 16 min. The column temperature was 45°C, and the flow rate was 0.3 ml/min. An HSS T3 chromatographic column (100 × 2.1 mm, 1.8 *μ*m, Waters, USA) was adopted. The system was equipped with an ESI source, and the detection conditions were under positive ion modes. The heater temperature was 320°C and the capillary temperature was 300°C, and the capillary voltage was 3.5 kV. Quercetin, formononetin, kaempferol, taxifolin, cryptotanshinone, and tanshinone IIA were identified as the main bioactive compounds using reference standards. The UPLC-MS analysis was performed using Xcalibur 2.2 software (Thermo Scientific, San Jose, USA).

### 2.15. Western Blot Analysis

Western blot procedures were performed as previously described [16]. The primary antibodies were rabbit anti-calcium/calmodulin-dependent protein kinase II (CaMKII) (1 : 1000, ab52476, Abcam, Cambridge, United Kingdom), rabbit anti-phospho- (P-) CaMKII (1 : 1000, ab5683, Abcam), rabbit anti-AKT (1 : 1000, #4685, Cell Signaling Technology, Danvers, MA, USA), rabbit anti-P-AKT (1 : 1000, #4060, Cell Signaling Technology), rabbit anti-P-ERK1/2 (1 : 1000, #4370, Cell Signaling Technology), rabbit anti-ERK1/2 (1 : 1000, #4695, Cell Signaling Technology), rabbit anti-P-p38 (1 : 1000, #9215, Cell Signaling Technology), rabbit anti-p38 (1 : 1000, #9212, Cell Signaling Technology), rabbit anti-P-SAPK/JNK (1 : 1000, #4668, Cell Signaling Technology), rabbit anti-SAPK/JNK (1 : 1000, #9258, Cell Signaling Technology), rabbit anti-TLR4 (1 : 1000, #14358, Cell Signaling Technology), rabbit anti-NF-*κ*B p65 (1 : 1000, #8242, Cell Signaling Technology), rabbit anti-P-NF-*κ*B p65 (1 : 1000, #3033, Cell Signaling Technology), and rabbit anti-*β*-actin (1 : 2000, #4970, Cell Signaling Technology). Horseradish peroxidase- (HRP-) conjugated goat anti-rabbit IgG (1 : 5000, A8275, Sigma-Aldrich) was used as a secondary antibody.

### 2.16. Mouse Cytokine Array

Serum samples were harvested from each group at 24 h after CLP surgery. For each sample, 60 *μ*l serum was used to determine the concentration of 20 cytokines including granulocyte-macrophage colony-stimulating factor (GM-CSF), interferon-gamma (IFN-*γ*), interleukin- (IL-) 1*α*, IL-1*β*, IL-2, IL-3, IL-4, IL-5, IL-6, IL-9, IL-10, IL-12, IL-13, IL-17, keratinocyte-derived chemokine (KC), monocyte chemoattractant protein-1 (MCP-1), macrophage colony-stimulating factor (MCSF), regulated upon activation normal T expressed and secreted (RANTES), tumor necrosis factor-*α* (TNF-*α*), and vascular endothelial growth factor (VEGF) using Quantibody Mouse Cytokine Array 1 (RayBiotech, Inc., Norcross, GA, USA) according to the manufacturer's instruction. The data were analyzed with RayBiotech cytokine antibody array software [17].

### 2.17. Statistical Analysis

Data were presented as means ± standard deviation (SD). Statistical analysis was performed using the GraphPad Prism 7 program (GraphPad, La Jolla, USA). One-way analysis of variance (ANOVA) was performed to compare the statistical differences of data among three or more groups. A *P* value of <0.05 was considered statistically significant.

## 3. Results

### 3.1. QX1 Formula Improved the Survival Outcome and Attenuated Cardiac Dysfunction in Septic Mice

**Figure 2:**
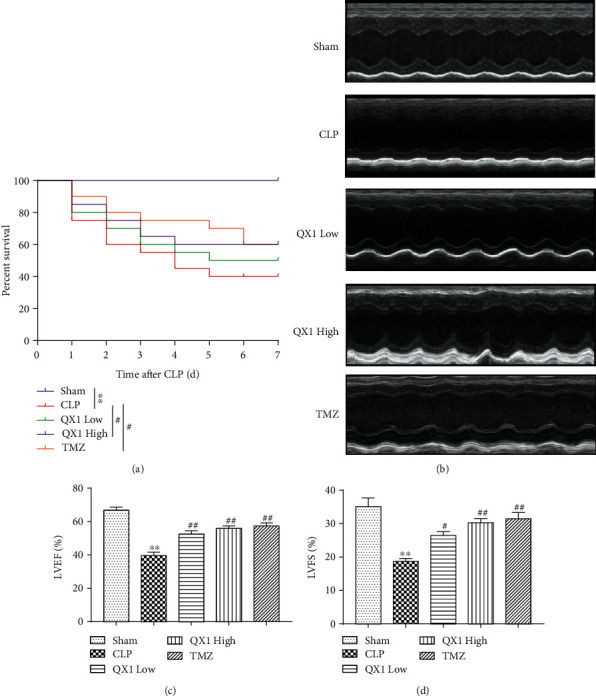
The QX1 formula improved the survival outcome and cardiac dysfunction in septic mice. Mice were orally administered with low (5 g/kg) or high (10 g/kg) dose of the QX1 formula or TMZ (20 mg/kg) at 6 h and 18 h after CLP surgery, respectively. (a) Kaplan-Meier survival curves. Twenty mice of each group were used to analyze the 7-day mortality. (b) Representative M-mode echocardiograms after CLP surgery. (c) Left ventricle ejection fraction (EF) and (d) fractional shortening (FS) were calculated. Data were presented as means ± SD, and differences between means were compared using one-way ANOVA with Tukey's multiple comparison test. ^∗∗^*P* < 0.01 compared to the sham group; ^#^*P* < 0.05, ^##^*P* < 0.01 compared to the CLP group.

We initially investigated whether administration of the QX1 formula conferred a survival advantage to septic mice (Figure 2(a)). The survival rate of CLP mice was approximately 55% within 3 days and 40% within 7 days. The Kaplan-Meier survival analysis showed that mice in the QX1 Low group exhibited an increased 7-day survival rate to 50% compared to the CLP mice. Both the high dose of the QX1 formula and TMZ treatment significantly improved the 7-day survival rate to 60% as compared with that of CLP mice (*P* < 0.05 and *P* < 0.05, respectively). Then, we performed echocardiography analysis to assess the effect of the QX1 formula on cardiac function in septic mice (Figure 2(b)). Echocardiography analysis found that mice that underwent CLP surgery had significantly reduced LVEF and LVFS compared with sham mice, while low or high dose of the QX1 formula significantly increased LVEF and LVFS (*P* < 0.05 and *P* < 0.01, respectively, Figures 2(c) and 2(d)). The LVEF and LVFS of mice in the TMZ group also significantly increased compared to those of mice in the CLP group (*P* < 0.05 and *P* < 0.05, respectively).

### 3.2. QX1 Formula Ameliorated the Disruption of Cardiac Structure in Septic Mice

**Figure 3:**
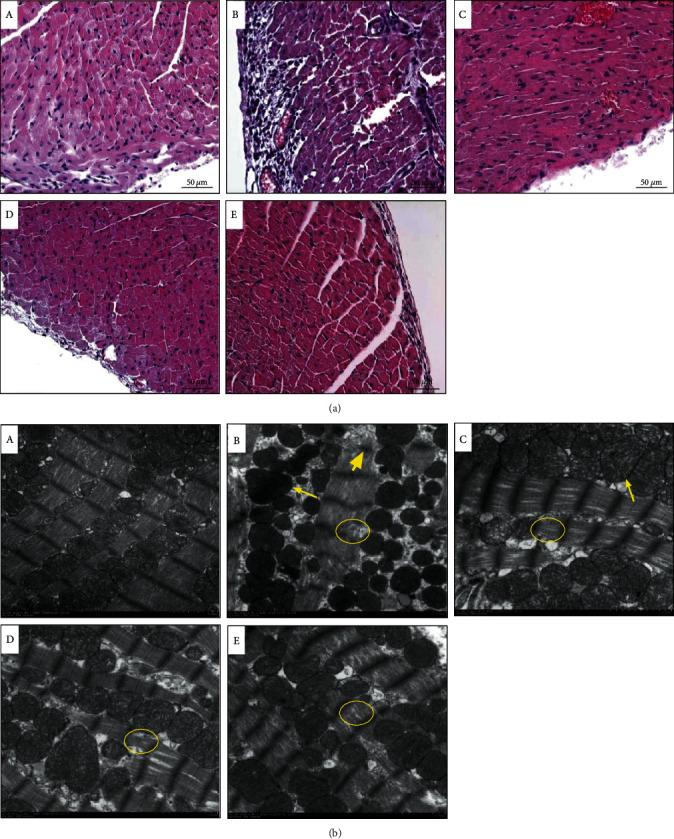
The QX1 formula ameliorated the disruption of cardiac structure in septic mice. (a) Representative H&E staining images of the left ventricular myocardium (scale bar = 50 *μ*m). (b) Representative images of transmission electron microscopy of the left ventricular myocardium (scale bar = 2 *μ*m). (A) Sham group, (B) CLP group, (C) QX1 Low group, (D) QX1 High group, and (E) TMZ group. The short yellow arrow indicated that the Z-line of the sarcomere was broken and blurred. The yellow circles indicated that myofibrils were loosely arranged and partially dissolved. The long yellow arrows indicated that mitochondria were swollen.

H&E staining was used to observe the effect of QX1 formula treatment on the pathological changes of cardiac tissue in septic mice (Figure 3(a)). The sham group showed normal histological features. In the CLP group, the cardiac structure was damaged, accompanied by a loose arrangement of myogenic fibers and inflammatory cell infiltration. Treatment with low or high dose of the QX1 formula or TMZ alleviated the loosening of cardiomyocytes and inflammatory cell infiltration. Further, the changes of cardiac structure were observed using TEM (Figure 3(b)). In the sham group, the myofibrils were organized orderly, the Z-line of the sarcomere was clear and straight, and the mitochondrial structure was completely arranged between the myofibrils. Compared with the sham group, in the CLP group, myofibril arrangement was loose and tortuous, with local dissolution and cavitation, the Z-line of the sarcomere was broken or blurred, the color of mitochondria became darker, and the arrangement was loose and swollen. In the QX1 Low group, myofibril arrangement was loose, the Z-line of the sarcomere was clear, and the mitochondria were swollen and proliferated. Compared with the CLP group, the myofibrils, Z-line, and mitochondrial structure were significantly improved in the QX1 High and TMZ groups.

### 3.3. Active Compound Screening

In this study, we initially obtained 513 chemical ingredients and their physicochemical properties from the QX1 formula from the TCMSP database and based on a large number of literatures. Then, the ADME system was applied to screen the potential active compounds of QX1. Finally, we screened 63 compounds which reached the standard of OB ≥ 28%, DL ≥ 0.18, HL ≥ 4, and Caco-2 cell permeability ≥ –0.4 as candidate active molecules (Table 1). There were 9, 10, 5, 11, and 31 active compounds in HQ, WWZ, FL, SHHZ, and DS, respectively. Among the active ingredients, quercetin (MOL02) and kaempferol (MOL15) were both in HQ and SHHZ, and hederagenin (MOL07) was found in both HQ and FL. On the basis of structure analysis, the 63 active compounds mainly belonged to diterpenoids, flavonoids, and lignans.

### 3.4. Drug Targeting and Analysis

In order to clarify the mechanism of QX1 active substances in the treatment of cardiac-related diseases, we need to clarify the possible targets of active compounds. A total of 79 potential targets for the 63 bioactive compounds were achieved using the WES algorithms and assigning them to the CTD, TTD, and PharmGKB databases (Supplementary Table 1). The results showed that most active compounds can act on multiple targets, and one target can be also possibly associated with multiple active compounds. The active compound quercetin (MOL02) can act on 56 targets, while the estrogen receptor (ESR1) was the target of 57 compounds, accounting for 90% of the total active compound targets.

### 3.5. Compound-Target Network Analysis

**Figure 4:**
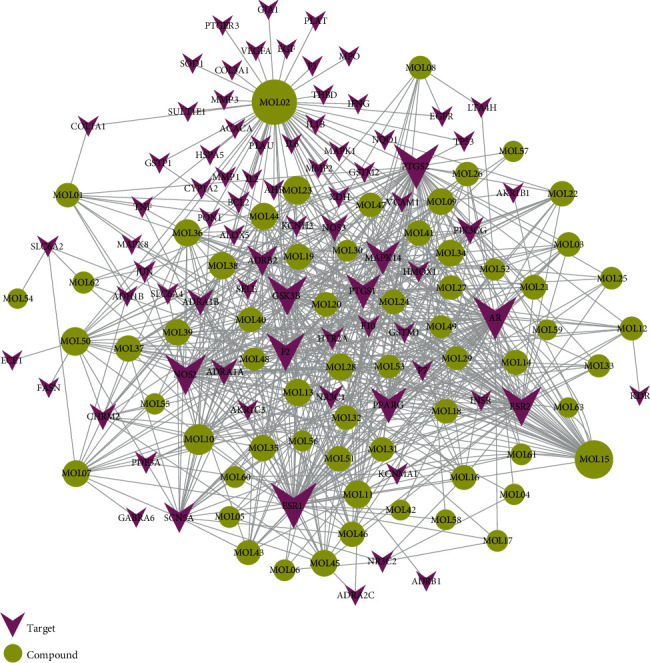
C-T network analysis. A compound node and a target node were connected if the protein was targeted by the corresponding compound. Node size was relative to its degree. The yellow circle represents the compounds, and the purple triangle represents the targets.

The C-T network diagram was constructed based on 142 nodes (63 potential compounds and 79 potential targets) and 686 edges (Figure 4). The degree parameter of topological analysis showed that the average degrees of potential compounds and targets were 10.9 and 8.7, respectively, indicating that the active compounds and targets were closely related in the QX1 formula. Quercetin (MOL02) is the key component of the QX1 formula and displayed the highest number of target interactions (degree = 56), followed by kaempferol (MOL15, degree = 33), beta-sitosterol (MOL10, degree = 24), tanshinone IIA (MOL50, degree = 19), formononetin (MOL13, degree = 16), cryptotanshinone (MOL35, degree = 14), and taxifolin (MOL21, degree = 11). Among potential protein targets, the top 10 high-degree targets acted on multiple compounds, namely, ESR1 (degree = 57), PTGS2 (degree = 55), AR (degree = 52), NOS2 (degree = 46), ESR2 (degree = 43), GSK3*β* (degree = 43), F2 (degree = 40), PPARG (degree = 35), PTGS1 (degree = 32), and MAPK14 (degree = 30) (Supplementary Table 2). These high-degree targets in the network may be the major mediators of the QX1 formula in the treatment of cardiac-related diseases.

### 3.6. Target-Protein Association Network Analysis

**Figure 5:**
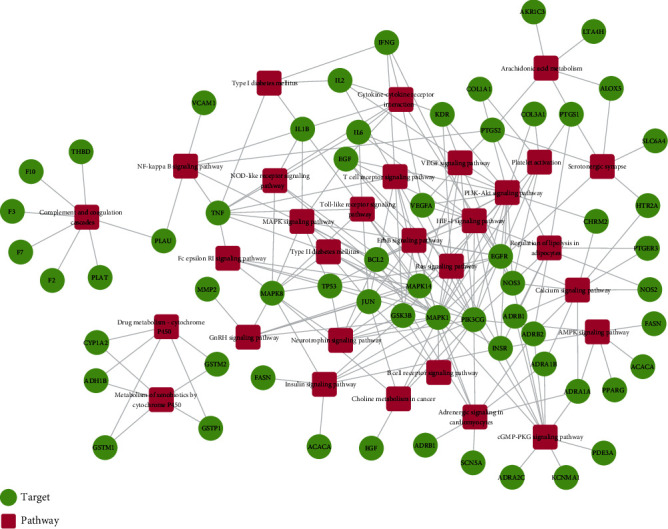
T-P network analysis. The T-P network was built by a target and a pathway if the pathway was lighted at the target. Node size was related to the degree. The green circle represents the target, and the red box represents the pathway.

The T-P network consists of 60 targets and 30 pathways significantly enriched by these targets (Figure 5). Obviously, most of the target proteins (40/60) appeared in multiple pathways, indicating that the target proteins of the QX1 formula interacted with each other in different pathways and carried out signal transmission for cardiac diseases. Meanwhile, many pathways (11/30) were also regulated by multiple target proteins (≥8), which might be the key mechanism of the QX1 formula in the treatment of cardiac-related diseases. As shown in Supplementary Table 3, the crucial target-protein associated pathways included the PI3K/AKT signaling pathway (degree = 16), HIF-1 signaling pathway (degree = 11), calcium signaling pathway (degree = 10), MAPK signaling pathway (degree = 9), cytokine-cytokine receptor interaction (degree = 9), adrenergic signaling in cardiomyocytes (degree = 9), Toll-like receptor signaling pathway (degree = 8), and T cell receptor signaling pathway (degree = 8).

### 3.7. Cardiac Disease-Related Pathway Analysis

**Figure 6:**
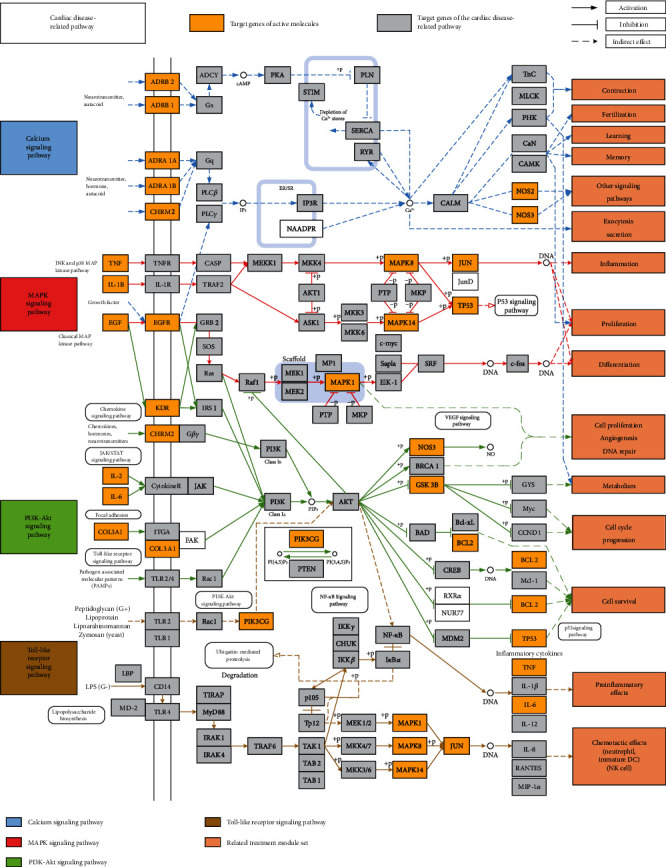
Distribution of target proteins of the QX1 formula on the integrated “cardiac disease-related pathway.” The cardiac disease-related pathway contained calcium, MAPK, PI3K/AKT, and Toll-like receptor signaling pathways. Arrows represent activation activity, T-arrows show inhibition activity, and segments represent indirect activation effect.

Considering the complex mechanism of QX1 in the treatment of cardiac-related diseases, an integrated map of “cardiac disease-related pathways” was constructed by integrating the key pathways that were obtained from the KEGG database and combined with T-P network analysis (Figure 6). The cardiac disease-related pathways were comprised of four important pathways: calcium signaling pathway, MAPK signaling pathway, PI3K/AKT signaling pathway, and TLR signaling pathway. As shown in Figure 6, the cardiac disease-related pathways were involved in several biological functions, such as contraction, inflammation, proliferation, differentiation, cell survival, cell cycle, and chemotactic effects. The QX1 formula may play a therapeutic role in cardiac disease by regulating these biological functions.

### 3.8. Target-Tissue Location Analysis

**Figure 7:**
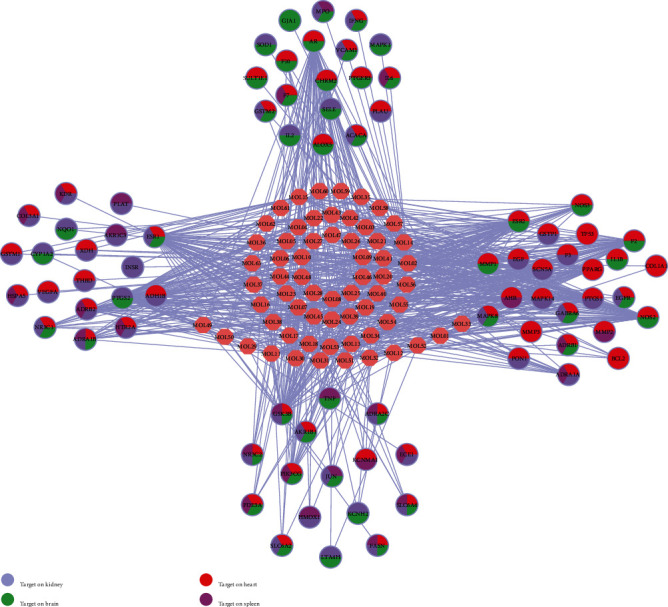
Tissue organ distribution of the target proteins of the QX1 formula. The pink node represents the compound molecule, and colored circles represent the target protein nodes and the organs where the target proteins are located.

Understanding the localization of protein targets on multiple organs at the system level is useful to clarify the therapeutic target of QX1 against cardiac functional diseases. A total of 79 targets were mapped on 84 normal tissues based on the BioGPS database. The tissue distribution network of the 79 targets was divided into heart, spleen, kidney, and brain tissue modules (Figure 7). Most targets acted on two or more tissues, which suggested that these tissues were closely correlated. Specifically, there were 62 targets that contained high mRNA expression in the heart, accounting for 78% of all the targets. Besides, 52 targets in the kidney, 47 targets in the brain, and 24 targets in the spleen were found, respectively. The results suggested that the target of the QX1 formula is closely linked to cardiac disease.

### 3.9. Identification of the Main Bioactive Compounds in Plasma

**Figure 8:**
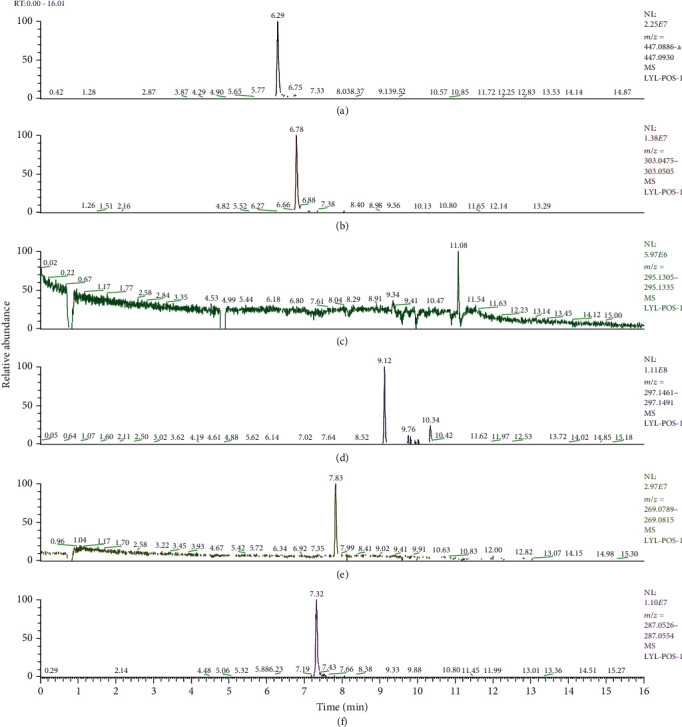
Chromatograms of the six main bioactive compounds in plasma at 30 min after oral administration of QX1: (a) taxifolin, (b) quercetin, (c) tanshinone IIA, (d) cryptotanshinone, (e) formononetin, and (f) kaempferol.

The six main bioactive compounds, including quercetin, formononetin, kaempferol, taxifolin, cryptotanshinone, and tanshinone IIA, were identified in plasma after oral administration of QX1 decoction by UPLC-MS. The chromatograms of the six main bioactive compounds at 30 min after oral administration of QX1 decoction are shown in Figure 8. The retention times were approximately 6.29 min for taxifolin, 6.78 min for quercetin, 11.08 min for tanshinone IIA, 10.34 min for cryptotanshinone, 7.83 min for formononetin, and 7.32 min for kaempferol. The chromatograms of analytes in blank plasma and blank plasma spiked with the six main bioactive compounds are shown in Supplementary Figure 1A and B.

### 3.10. Effect of the QX1 Formula on the Activity of Cardiac Disease-Related Pathways

**Figure 9:**
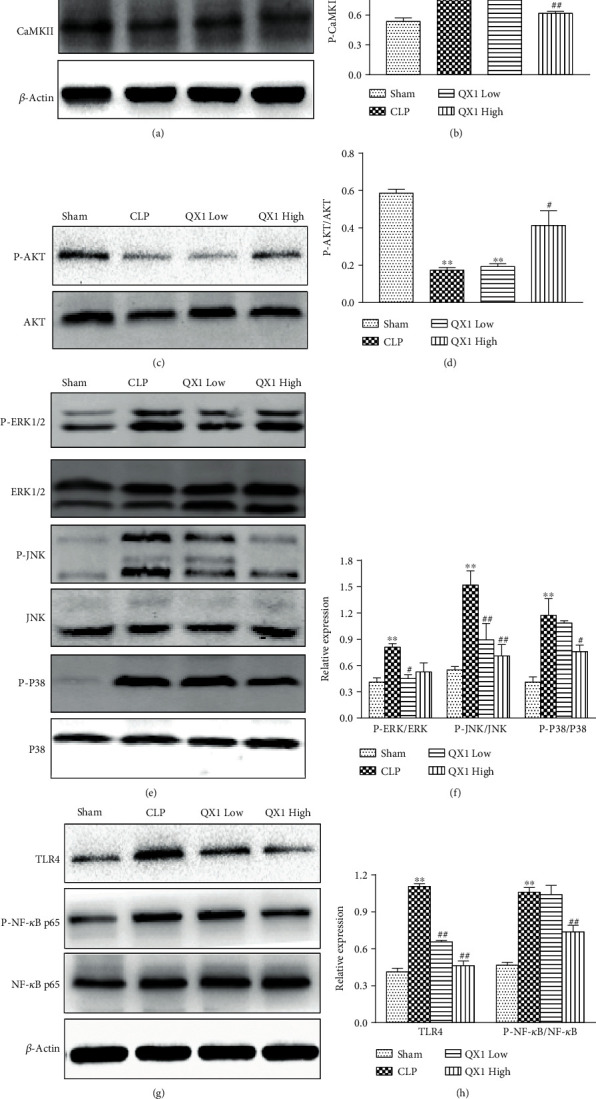
Effects of the QX1 formula on the sepsis-induced cardiac dysfunction pathway. (a, b) Western blot analysis of total CaMKII and P-CaMKII protein expression in heart tissues. (c, d) The expression levels of P-AKT and AKT proteins were determined in cardiac tissue. (e, f) Protein levels of ERK1/2, P-ERK1/2, P38, P-P38, JNK, and P-JNK were detected by Western blot. (g, h) Protein levels of TLR4 and NF-*κ*B were detected by Western blot. Data were presented as means ± SD, and differences between means were compared using one-way ANOVA with Tukey's multiple comparison test. ^∗∗^*P* < 0.01 compared to the sham group, ^#^*P* < 0.05, ^##^*P* < 0.01 compared to the CLP group.

In order to evaluate the consequences of systematic pharmacological analysis, we examined the effect of the QX1 formula on key proteins in the integrated “cardiac disease-related pathways,” including calcium, MAPK, PI3K/AKT, and TLR4 signaling pathways using Western blot. CLP surgery significantly increased the expression of P-CaMKII protein in the cardiac tissue of mice compared with the sham group, while low or high dose of QX1 formula treatment inhibited this increase (Figures 9(a) and 9(b)). Compared with the sham group, expression of P-AKT protein was decreased in the CLP group (Figures 9(c) and 9(d)). Compared with the CLP group, expression of P-AKT protein was increased in the QX1 High group, but not in the QX1 Low group. Furthermore, we investigated the effect of the QX1 formula on the activity of three well-characterized subfamilies of MAPK pathways, ERK1/2, JNK, and p38 (Figures 9(e) and 9(f)). CLP surgery induced the activation of ERK1/2, JNK, and P38 compared with the sham group, whereas the CLP-induced activation of JNK was inhibited by low or high dose of QX1 formula treatment. Compared with the CLP group, decreased expression of P-ERK1/2 and P-P38 was observed in the QX1 Low group and QX1 High group, respectively. In addition, the activity of the TLR4 pathway was also examined. CLP treatment significantly increased the expression of TLR4, and this increase was inhibited by low or high dose of QX1 formula treatment (Figures 9(g) and 9(h)). Compared with the sham group, increased expression of P-NF-*κ*B p65 downstream of TLR4 was observed in the CLP group, whereas this increase was inhibited by high but not low dose of QX1 formula treatment.

### 3.11. Effect of the QX1 Formula on Serum Cytokine Production

**Figure 10:**
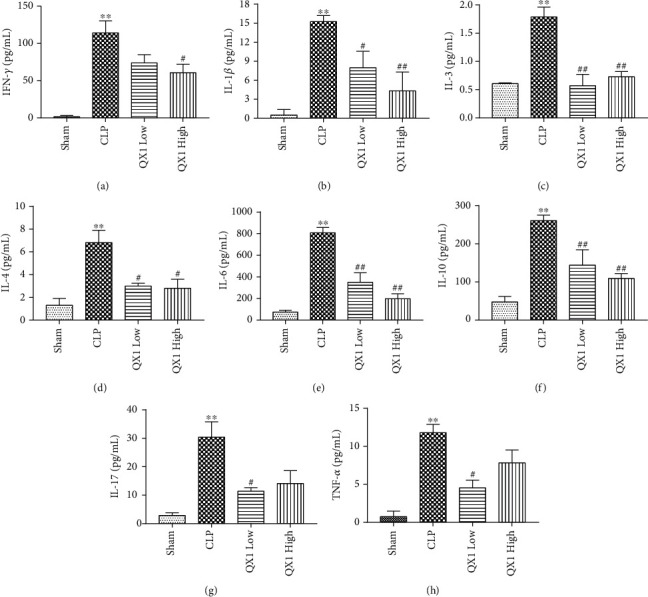
Effects of the QX1 formula on cytokine expression. The levels of (a) IFN-*γ*, (b) IL-1*β*, (c) IL-3, (d) IL-4, (e) IL-6, (f) IL-10, (g) IL-17, and (h) TNF-*α* in serum were quantified by a mouse cytokine assay. Data were presented as means ± SD, and differences between means were compared using one-way ANOVA with Tukey's multiple comparison test. ^∗∗^*P* < 0.01 compared to the sham group. ^#^*P* < 0.05, ^##^*P* < 0.01 compared to the CLP group.

Cytokines have been thought to play an important role in the induction of cardiac dysfunction during sepsis. The production of 20 cytokines in serum of mice was determined by a multiplex assay after CLP or QX1 formula treatment (Figure 10). Compared with the sham group, the levels of eight cytokines (IFN-*γ*, IL-1*β*, IL-3, IL-4, IL-6, IL-10, IL-17, and TNF-*α*) were significantly upregulated in the CLP group. Among them, IL-1*β*, IL-3, IL-4, IL-6, and IL-10 increased in the CLP group were markedly reduced in both the QX1 Low and QX1 High groups. Compared with the CLP group, the levels of IL-17 and TNF-*α* were reduced in the QX1 Low group and the level of IFN-*γ* was reduced in the QX1 High group.

## 4. Discussion

In this study, we demonstrated that the QX1 formula improved the survival outcome and ameliorated cardiac dysfunction in septic mice induced by CLP surgery. Based on the complex multicomponent property of the QX1 formula, a systems pharmacology approach was applied to explore the potential active components, targets, and networks. After *in silico* TCMSP-based prediction, we performed Western blot and mouse cytokine array experiments to verify our predicted pathway and elucidated the preliminary mechanism. We found that QX1 formula treatment enhanced the activation the PI3K/AKT pathway and attenuated the activity of the calcium, MAPK, and TLR4/NF-*κ*B pathway in the septic mice. To our knowledge, this is the first report to comprehensively elucidate the protective mechanism of the QX1 formula on sepsis-induced cardiac dysfunction.

Cardiac dysfunction is a common complication in patients with sepsis and dramatically increases mortality from 20% to as high as 70%–90% in patients with sepsis [18, 19]. CLP surgery in mice is the most frequently used experimental model and is considered the gold standard in sepsis research [20]. The position of cecal ligation in mice is the primary determinant of sepsis severity and mortality. Reduction of sepsis mortality is one of the most important indicators to evaluate the efficacy of drug therapy. The QX1 formula is an applicable TCM prescription for sepsis-related cardiac dysfunction and has been used in the clinical practice for more than 30 years. Our previous study showed that the high-dose QX1 formula significantly increased the 3-day survival rate in mice with severe sepsis from 22% to 40% [7]. In the present study, a CLP-induced moderate sepsis model was established and 60% of mice died during 7 days. Administration of low (5 g/kg) or high dose of QX1 (10 g/kg) improved the survival outcome in septic mice and led to an increase in the 7-day survival rate to 50% and 60%, respectively. Echocardiography is the most effective tool to evaluate the cardiac function of sepsis. The LVEF and LVFS are well-known powerful factors for predicting the mortality and outcome in heart failure patients [21, 22]. We found that QX1 formula treatment notably elevated LVEF and LVFS in septic mice. Moreover, QX1 formula treatment alleviated the sepsis-induced damage of cardiac histological and ultrastructure. The effects of the high-dose QX1 formula on the survival outcome, LVEF and LVFS, and cardiac morphological structure damage were comparable to those of TMZ treatment. Our results suggested that administration of the QX1 formula effectively improved the survival outcome and ameliorated sepsis-induced cardiac dysfunction.

Deeply studying the molecular mechanism of TCM is difficult due to its multicomponent property. Now, systems pharmacology has become a promising approach to elucidate the mechanisms of multiple target components in TCM [23]. Using the ADME system, 63 QX1 potential active compounds were screened out in this study based on the standards of OB ≥ 28%, DL ≥ 0.18, HL ≥ 4, and Caco-2 cell permeability ≥ –0.4. Among them, quercetin (MOL02), formononetin (MOL13), kaempferol (MOL15), taxifolin (MOL21), cryptotanshinone (MOL35), and tanshinone IIA (MOL50) are also identified and quantified using UPLC-MS/MS analysis in our previous study, which confirmed the reliability of systematic pharmacological screening of herbal active ingredients [7]. In the present study, these six active compounds were also detectable in rat plasma after treatment of QX1 decoction. QX1 formula treatment ameliorates myocardial tissue damage in mice suffering from sepsis partly by inhibiting endoplasmic reticulum- and mitochondria-related apoptosis [7]. In this study, as predicted by a systems pharmacology approach, QX1 may play a therapeutic role in sepsis-induced cardiac dysfunction primarily by regulating calcium signaling, MAPK, PI3K/AKT, and TLR pathways. To further validate this prediction, we evaluated the effect of QX1 on the key protein expression in these pathways in septic mice using Western blot. QX1 formula treatment significantly inhibited the sepsis-induced activation of CaMKII, MAPK (P38, ERK1/2, and JNK), and TLR4/NF-*κ*B pathways and promoted the activation of AKT. This study proved the reliability of the systems pharmacology approach in exploring cardiac protective effect and the underlying mechanism of QX1.

Accumulating evidence has documented that calcium signaling plays a pivotal role in sepsis-induced cardiac dysfunction [24, 25]. CaMKII is a molecular switch that regulates myocardial Ca^2+^ signaling, and excessive CaMKII activation is detrimental to the integrity and function of the heart [26, 27]. The activity of CaMKII was significantly increased in septic mice [25]. In this study, QX1 treatment decreased the level of P-CaMKII. Cryptotanshinone (degree = 14) and tanshinone IIA (degree = 19), the primary bioactive compounds in Danshen, ameliorate hypoxia-induced damage of cardiomyocyte H9c2 cells by regulating intracellular NO, calcium, and mitochondrial ROS production [24]. It was proposed that the bioactive compounds cryptotanshinone and tanshinone in the QX1 formula may alter Ca^2+^ handling to exert their cardiac protective effects.

The PI3K/AKT pathway is a classical pathway that regulates cell proliferation, survival, and cell homeostasis [28]. The previous study has shown that inhibition of PI3K increased the inflammatory and apoptotic processes and mortality in septic mice [29]. By contrast, the activation of the PI3K/AKT pathway improved cardiac dysfunction and reduced sepsis mortality in an animal sepsis model [30, 31]. Formononetin (degree = 16), a methoxyisoflavone widely found in many herbs, has been shown to protect cardiomyocyte H9c2 cells from oxygen-glucose deprivation and reoxygenation injury via suppression of reactive oxygen species (ROS) formation by promoting AKT activation and GSK-3*β* phosphorylation [32]. Quercetin, a natural flavonoid, is the key component of QX1 and displayed the highest number of target interactions (degree = 56). Besides, quercetin postconditioning significantly alleviates cardiac ischemia/reperfusion injury in rats via activating the PI3K/AKT pathway [33]. Our study demonstrated that formononetin and quercetin in the QX1 formula may activate the PI3K/Akt pathway, which partially contributes to their curative effects.

MAPK, as the serine-threonine kinases, regulates several important cellular processes, including cell proliferation, inflammation, survival, stress response, and apoptosis [34]. A recent study revealed that inhibition of MAPK signaling pathways could alleviate sepsis-induced cardiac injury in AT1R-knockdown rats [35]. ERK1/2, JNK, and p38 are the three major subfamilies of MAPK signaling proteins. Taxifolin (degree = 11), an active flavonoid, was shown to exert a cardioprotective effect against cardiac ischemia/reperfusion injury by modulating oxidative stress and attenuating mitochondrial apoptosis [36]. Kaempferol (degree = 33), a dietary flavonoid, has been indicated to ameliorate myocardial ischemic injury by inhibiting the phosphorylation of JNK and p38 proteins and activation of ERK1/2 [37], which may be responsible for the inhibitory effect of the QX1 formula on the MAPK pathway in septic mice.

In sepsis, the activation of the MAPK pathway might result from aberrant upstream signaling, such as TLR4 [38]. TLR4 is one of the most studied members of the TLR family, which plays a pivotal role in the signal transduction of sepsis-induced inflammatory response. It has been reported that activation of TLR4 induces inflammation and aggravates cardiac dysfunction in severe sepsis, while knockout of the *TLR4* gene improves sepsis-induced cardiac dysfunction [39]. Therefore, TLR4 has been considered a potential therapeutic target for controlling inflammatory response and improving cardiac function [40]. The NF-*κ*B pathway, a typical inflammatory signaling pathway, can be activated by TLR4 and lead to the excessive release of proinflammatory cytokines leading to secondary sepsis myocardial injury [41]. In the present study, we found that the TLR4/NF-*κ*B signaling pathway was activated during sepsis. QX1 formula treatment significantly inhibited the activation of the TLR4/NF-*κ*B inflammatory signaling pathway. It was reported that in mice, quercetin protects mice from LPS-induced sepsis by inhibiting proinflammatory cytokine TNF-*α* and IL-1*β* expression, NF-*κ*B activation, and apoptosis [42]. Quercetin in the QX1 formula may play an important role in preventing myocardial dysfunction via the TLR4/NF-*κ*B signaling pathway during sepsis.

An acute severe systemic inflammatory response known as “cytokine storm” is a key factor in the development and progression of septic cardiac dysfunction [43]. Both proinflammatory and opposing anti-inflammatory responses occur concomitantly in sepsis, and sepsis is regarded as an immunosuppressive disorder [44]. Analysis of cytokine profiles and mortality in 464 patients showed that a high ratio of IL-10 to TNF-*α* is associated with mortality in patients with community-acquired infection [44]. In this study, we observed that sepsis led to cytokine storm accompanied by the upregulated serum levels of IFN-*γ*, IL-1*β*, IL-3, IL-4, IL-6, IL-10, IL-17, and TNF-*α*, whereas QX1 treatment decreased the production of typical Th1/Th2-associated proinflammatory cytokines (IFN-*γ*, IL-1*β*, IL-3, IL-6, and TNF-*α*) and Th17-associated proinflammatory cytokines (IL-17). Our study also found that QX1 markedly downregulated the levels of typical Th2-associated anti-inflammatory cytokines (IL-4, IL-10). Elevated concentrations of TNF-*α* and IL-1*β* are found in the serum of septic patients and are responsible for sepsis-related cardiac depression [45]. The IL-1*β* level is also increased in LPS-treated mice and plays an important role in suppressing myocardial contractility [46]. TNF-*α* is a proinflammatory cytokine mainly expressed in the initial hyperinflammatory stage of sepsis and is responsible for myocardial diastolic and systolic dysfunction [47]. In sepsis, overexpression of TNF-*α* increases the level of NO by inducing the production of inducible nitric oxide synthase (iNOS), which leads to apoptosis of myocardial cells and heart failure [48]. Suppression of the systolic function of cardiomyocytes *in vitro* is associated with IL-6 production, and removal of IL-6 in the culture supernatant significantly improves the systolic function of cardiomyocytes [49]. IL-3 plays a critical role during sepsis. It was reported that the addition of a CD123 (IL-3 receptor alpha chain) antibody reduces mortality and alleviates organ dysfunction by restraining the JAK2-STAT5 signaling pathway and reduces serum cytokines in the development of early sepsis in a rat model induced by CLP [50]. IL-6 contributes to host defense against infections and tissue injuries; however, excessive levels of IL-6 lead to cytokine storm via inhibiting cardiac function but activating the coagulation pathway and vascular endothelial cells [51]. In the CLP-induced sepsis, calcium-sensing receptor activation promotes T cell apoptosis and the secretion of the proinflammatory cytokine TNF-*α* and the anti-inflammatory cytokine IL-4 probably through NF-*κ*B and partial ERK and JNK signal transduction pathways [52]. Our results suggested that the QX1 formula may constitute a novel therapeutic strategy for suppressing the activity of CaMKII, TLR4/NF-*κ*B, and MAPK pathways, but promoting the activation of AKT, thereby decreasing the release of downstream inflammatory cytokines and thus controlling cytokine storm and regulating immune balance in sepsis.

In conclusion, the QX1 formula improved cardiac dysfunction in sepsis mice by inhibiting calcium, MAPK, and TLR4 signaling pathways, activating PI3K/AKT pathways, and reducing the subsequent release of inflammation cytokines. This study demonstrated the multicomponent, multitarget, and multipathway characteristics of QX1, which provided a novel understanding of QX1 in the clinical application on cardiac dysfunction during sepsis.

## Figures and Tables

**Table 1:** Active compounds and their corresponding ADME parameters in the QX1 formula.

Molecular ID	Compounds	Herb	OB	Caco-2	DL	HL	Degree	Structure
MOL01	Palmitic acid	FL	19.30	1.09	0.10	0.00	10	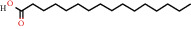
MOL02	Quercetin	HQ/SHHZ	46.43	0.05	0.28	14.40	56	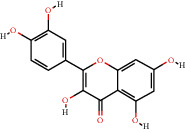
MOL03	Jaranol	HQ	50.83	0.61	0.29	15.50	10	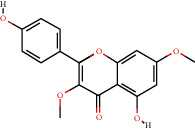
MOL04	(2R)-2-[(3S,5R,10S,13R,14R,16R,17R)-3,16-Dihydroxy-4,4,10,13,14-pentamethyl-2,3,5,6,12,15,16,17-octahydro-1H-cyclopenta[a]phenanthren-17-yl]-6-methylhept-5-enoic acid	FL	30.93	0.01	0.81	6.81	3	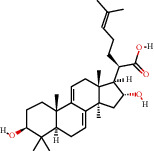
MOL05	Trametenolic acid	FL	38.71	0.52	0.80	7.78	4	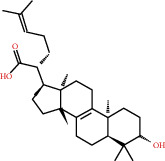
MOL06	Cerevisterol	FL	37.96	0.28	0.77	5.31	4	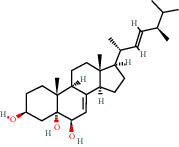
MOL07	Hederagenin	HQ/FL	36.91	1.32	0.75	5.35	15	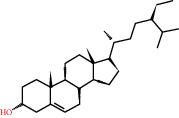
MOL08	n-Coumaroyltyramine	SHHZ	85.63	0.69	0.20	4.82	8	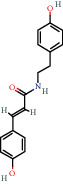
MOL09	Isorhamnetin	HQ	49.60	0.31	0.31	14.34	15	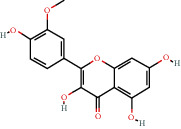
MOL10	Beta-sitosterol	SHHZ	36.91	1.32	0.75	5.36	24	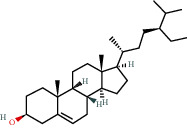
MOL11	3,9-Di-O-methylnissolin	HQ	53.74	1.18	0.48	9.00	17	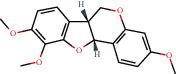
MOL12	Bifendate	HQ	31.10	0.15	0.67	17.96	9	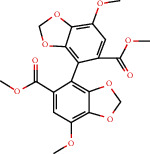
MOL13	Formononetin	HQ	69.67	0.78	0.21	17.04	16	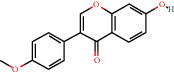
MOL14	Calycosin	HQ	47.75	0.52	0.24	17.10	11	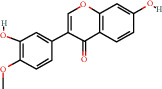
MOL15	Kaempferol	HQ/SHHZ	41.88	0.26	0.24	14.74	41	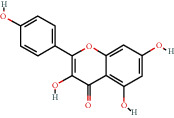
MOL16	(2R)-5,7-Dihydroxy-2-(4-hydroxyphenyl)chroman-4-one	SHHZ	42.36	0.38	0.21	16.83	11	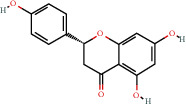
MOL17	Poriferasterol	DS	43.83	1.44	0.76	5.34	4	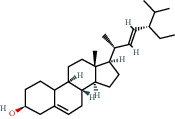
MOL18	(-)-Taxifolin	SHHZ	60.51	-0.24	0.27	14.37	10	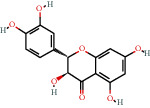
MOL19	Dehydrotanshinone II A	DS	43.76	1.02	0.40	23.71	13	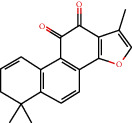
MOL20	Chryseriol	SHHZ	35.85	0.39	0.27	16.31	10	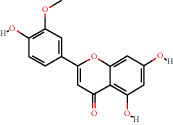
MOL21	Taxifolin	SHHZ	57.84	-0.23	0.27	14.41	11	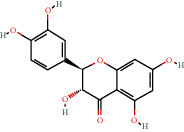
MOL22	Eriodictyol	SHHZ	71.79	0.17	0.24	15.81	12	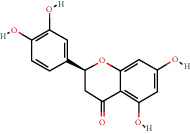
MOL23	2-Isopropyl-8-methylphenanthrene-3,4-dione	DS	40.86	1.23	0.23	14.89	19	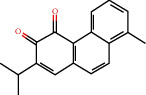
MOL24	3*α*-HydroxytanshinoneIIa	DS	44.93	0.53	0.44	23.78	10	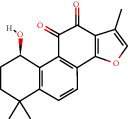
MOL25	(E)-3-[2-(3,4-Dihydroxyphenyl)-7-hydroxy-benzofuran-4-yl]acrylic acid	DS	48.24	0.18	0.31	8.87	6	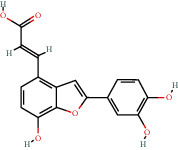
MOL26	Formyltanshinone	DS	73.44	0.54	0.42	24.12	10	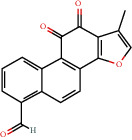
MOL27	Przewaquinone B	DS	62.24	0.39	0.41	24.94	10	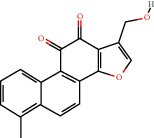
MOL28	Przewaquinone C	DS	55.74	0.42	0.40	23.70	15	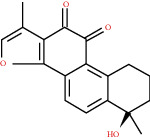
MOL29	Przewaquinone F	DS	40.31	-0.09	0.46	22.45	8	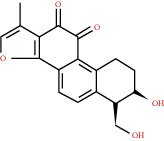
MOL30	Sclareol	DS	43.67	0.84	0.21	4.71	4	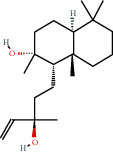
MOL31	Tanshinaldehyde	DS	52.47	0.57	0.45	23.49	10	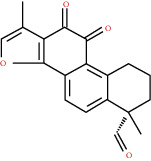
MOL32	Tanshinol A	DS	21.31	0.36	0.41	0.00	10	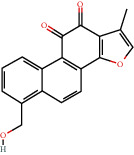
MOL33	Danshenol B	DS	57.95	0.53	0.56	4.28	7	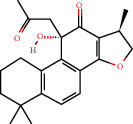
MOL34	Danshenol A	DS	56.97	0.33	0.52	5.15	15	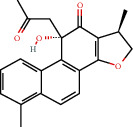
MOL35	Cryptotanshinone	DS	52.34	0.95	0.40	17.30	14	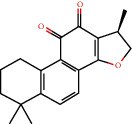
MOL36	Danshenspiroketallactone	DS	50.43	0.88	0.31	15.19	16	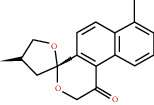
MOL37	Deoxyneocryptotanshinone	DS	49.40	0.85	0.29	27.17	14	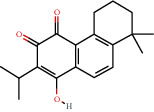
MOL38	Dihydrotanshinone I	DS	45.04	0.95	0.36	18.32	16	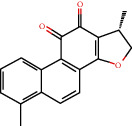
MOL39	Isocryptotanshinone	DS	54.98	0.93	0.39	31.92	14	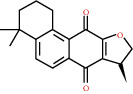
MOL40	Isotanshinone II	DS	49.92	1.03	0.40	24.73	11	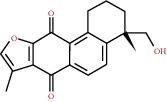
MOL41	Isotanshinone I	DS	29.72	1.01	0.36	0.00	12	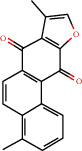
MOL42	Manool	DS	45.04	1.28	0.20	5.81	2	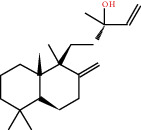
MOL43	Methyltanshinonate	DS	19.19	0.56	0.55	0.00	11	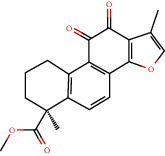
MOL44	Miltionone I	DS	49.68	0.35	0.32	41.49	16	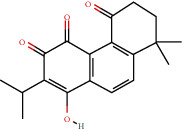
MOL45	Miltirone	DS	38.76	1.23	0.25	14.82	15	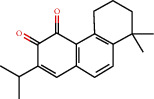
MOL46	Neocryptotanshinone	DS	52.49	0.35	0.32	14.46	12	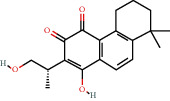
MOL47	Prolithospermic acid	DS	64.37	0.10	0.31	8.82	10	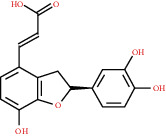
MOL48	Tanshindiol B	DS	42.67	0.05	0.45	22.25	7	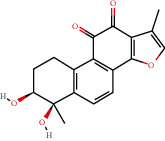
MOL49	Przewaquinone E	DS	42.85	-0.04	0.45	22.44	7	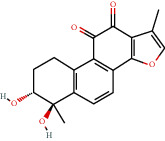
MOL50	Tanshinone IIa	DS	49.89	1.05	0.40	23.56	19	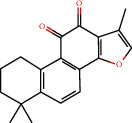
MOL51	Tanshinone VI	DS	45.64	0.48	0.30	15.21	12	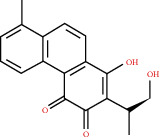
MOL52	2-(4-Hydroxyphenyl)ethyl (E)-3-(4-hydroxyphenyl)prop-2-enoate	SHHZ	93.36	0.68	0.21	5.24	5	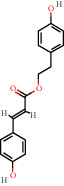
MOL53	Schisanhenol	WWZ	22.98	1.88	0.06	0.00	8	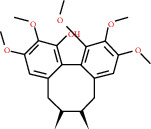
MOL54	4,7-Dimethyl-7-(4-methylpent-3-enyl)bicyclo[2.2.1]heptan-3-ol	WWZ	30.71	0.66	0.83	9.40	1	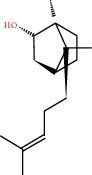
MOL55	Angeloylgomisin H	WWZ	29.70	1.83	0.09	0.00	3	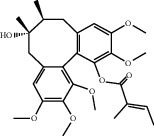
MOL56	Schizandrer B	WWZ	25.37	0.10	0.04	0.00	2	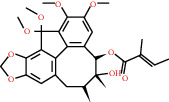
MOL57	Clupanodonic acid	WWZ	30.69	0.63	0.78	5.09	3	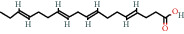
MOL58	Gomisin D	WWZ	32.68	0.73	0.83	8.50	2	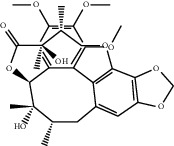
MOL59	Gomisin H	WWZ	34.84	0.60	0.86	9.54	2	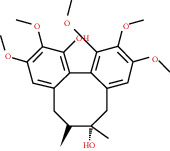
MOL60	Schisanhenol acetate	WWZ	27.20	1.86	0.02	0.00	4	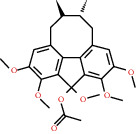
MOL61	Schizonepetoside A	WWZ	48.80	1.39	0.03	11.35	4	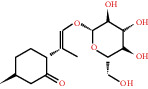
MOL62	Thuja alcohol	WWZ	46.27	1.08	0.84	8.72	5	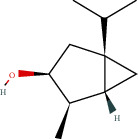
MOL63	Kaempferol-3-O-*α*-L-rhamnoside	SHHZ	41.88	-1.29	0.69	16.15	1	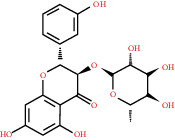

## Data Availability

The data used to support the findings of this study are available from the corresponding authors upon request.

## Supplementary Materials

Supplementary 1 Figure S1: chromatograms of the six main bioactive compounds in rat plasma. (A) Blank plasma. (B) Blank rat plasma spiked with the six main bioactive compounds: (a) taxifolin, (b) quercetin, (c) tanshinone IIA, (d) cryptotanshinone, (e) formononetin, and (f) kaempferol.

Supplementary 2 Table S1: the potential targets for the 63 bioactive compounds in the QX1 formula.

Supplementary 3 Table S2: the degree of protein targets corresponding to potential compounds in the QX1 formula.

Supplementary 4 Table S3: the information of the target-pathway network in the QX1 formula.

## References

[1] Singer M., Deutschman C. S., Seymour C. W. (2016). The third international consensus definitions for sepsis and septic shock (Sepsis-3). *Journal of the American Medical Association*.

[2] Prescott H. C., Angus D. C. (2018). Enhancing recovery from sepsis: a review. *Journal of the American Medical Association*.

[3] Zheng Z., Ma H., Zhang X. (2017). Enhanced glycolytic metabolism contributes to cardiac dysfunction in polymicrobial sepsis. *The Journal of Infectious Diseases*.

[4] Arfaras-Melainis A., Polyzogopoulou E., Triposkiadis F. (2020). Heart failure and sepsis: practical recommendations for the optimal management. *Heart Failure Reviews*.

[5] Liu Y. C., Yu M. M., Shou S. T., Chai Y. F. (2017). Sepsis-induced cardiomyopathy: mechanisms and treatments. *Frontiers in Immunology*.

[6] Jiang W., Li W., Hu X., Hu R., Li B., Lan L. (2020). CTRP1 prevents sepsis-induced cardiomyopathy via Sirt1-dependent pathways. *Free Radical Biology & Medicine*.

[7] Xu X., Liu Q., He S. (2018). Qiang-Xin 1 formula prevents sepsis-induced apoptosis in murine cardiomyocytes by suppressing endoplasmic reticulum- and mitochondria-associated pathways. *Frontiers in Pharmacology*.

[8] Zhang W., Huai Y., Miao Z., Qian A., Wang Y. (2019). Systems pharmacology for investigation of the mechanisms of action of traditional Chinese medicine in drug discovery. *Frontiers in Pharmacology*.

[9] Lv W. J., Liu C., Li Y. F. (2019). Systems pharmacology and microbiome dissection of Shen Ling Bai Zhu San reveal multiscale treatment strategy for IBD. *Oxidative Medicine and Cellular Longevity*.

[10] Su X., Li Y., Jiang M. (2019). Systems pharmacology uncover the mechanism of anti-non-small cell lung cancer for Hedyotis diffusa Willd. *Biomedicine & Pharmacotherapy*.

[11] Yu G., Luo Z., Zhou Y. (2019). Uncovering the pharmacological mechanism of Carthamus tinctorius L. on cardiovascular disease by a systems pharmacology approach. *Biomedicine & Pharmacotherapy*.

[12] Rittirsch D., Huber-Lang M. S., Flierl M. A., Ward P. A. (2009). Immunodesign of experimental sepsis by cecal ligation and puncture. *Nature Protocols*.

[13] Ru J., Li P., Wang J. (2014). TCMSP: a database of systems pharmacology for drug discovery from herbal medicines. *Journal of Cheminformatics*.

[14] Liu H., Wang J., Zhou W., Wang Y., Yang L. (2013). Systems approaches and polypharmacology for drug discovery from herbal medicines: an example using licorice. *Journal of Ethnopharmacology*.

[15] Zheng C., Guo Z., Huang C. (2015). Large-scale direct targeting for drug repositioning and discovery. *Scientific Reports*.

[16] He S., Liu F., Xu L. (2016). Protective effects of ferulic acid against heat stress-induced intestinal epithelial barrier dysfunction in vitro and in vivo. *PLoS One*.

[17] Xie S. Z., Liu B., Ye H. Y. (2019). Dendrobium huoshanense polysaccharide regionally regulates intestinal mucosal barrier function and intestinal microbiota in mice. *Carbohydrate Polymers*.

[18] Pulido J. N., Afessa B., Masaki M. (2012). Clinical spectrum, frequency, and significance of myocardial dysfunction in severe sepsis and septic shock. *Mayo Clinic Proceedings*.

[19] Fleischmann C., Scherag A., Adhikari N. K. (2016). Assessment of global incidence and mortality of hospital-treated sepsis. Current estimates and limitations. *American Journal of Respiratory and Critical Care Medicine*.

[20] Dejager L., Pinheiro I., Dejonckheere E., Libert C. (2011). Cecal ligation and puncture: the gold standard model for polymicrobial sepsis?. *Trends in Microbiology*.

[21] Aoyama D., Miyazaki S., Hasegawa K. (2020). Preprocedural troponin T levels predict the improvement in the left ventricular ejection fraction after catheter ablation of atrial fibrillation/flutter. *Journal of the American Heart Association*.

[22] Sun Y. P., Wei C. P., Ma S. C. (2015). Effect of carvedilol on serum heart-type fatty acid-binding protein, brain natriuretic peptide, and cardiac function in patients with chronic heart failure. *Journal of Cardiovascular Pharmacology*.

[23] Lee W. Y., Lee C. Y., Kim Y. S., Kim C. E. (2019). The methodological trends of traditional herbal medicine employing network pharmacology. *Biomolecules*.

[24] Jin H. J., Li C. G. (2013). Tanshinone IIA and cryptotanshinone prevent mitochondrial dysfunction in hypoxia-induced H9c2 cells: association to mitochondrial ROS, intracellular nitric oxide, and calcium levels. *Evidence-based Complementary and Alternative Medicine*.

[25] Sepulveda M., Gonano L. A., Viotti M. (2017). Calcium/calmodulin protein kinase II-dependent ryanodine receptor phosphorylation mediates cardiac contractile dysfunction associated with sepsis. *Critical Care Medicine*.

[26] Joiner M.-l. A., Koval O. M., Li J. (2012). CaMKII determines mitochondrial stress responses in heart. *Nature*.

[27] Beckendorf J., van den Hoogenhof M., Backs J. (2018). Physiological and unappreciated roles of CaMKII in the heart. *Basic Research in Cardiology*.

[28] Cantley L. C. (2002). The phosphoinositide 3-kinase pathway. *Science*.

[29] Williams D. L., Li C., Ha T. (2003). Modulation of the phosphoinositide 3-kinase pathway alters innate resistance to polymicrobial sepsis. *Journal of Immunology*.

[30] An R., Zhao L., Xi C. (2016). Melatonin attenuates sepsis-induced cardiac dysfunction via a PI3K/Akt-dependent mechanism. *Basic Research in Cardiology*.

[31] Li C., Hua F., Ha T. (2012). Activation of myocardial phosphoinositide-3-kinase p110*α* ameliorates cardiac dysfunction and improves survival in polymicrobial sepsis. *PLoS One*.

[32] Cheng Y., Xia Z., Han Y., Rong J. (2016). Plant Natural Product Formononetin Protects Rat Cardiomyocyte H9c2 Cells against Oxygen Glucose Deprivation and Reoxygenation via Inhibiting ROS Formation and Promoting GSK-3*β* Phosphorylation. *Oxidative Medicine and Cellular Longevity*.

[33] Wang Y., Zhang Z. Z., Wu Y., Ke J. J., He X. H., Wang Y. L. (2013). Quercetin postconditioning attenuates myocardial ischemia/reperfusion injury in rats through the PI3K/Akt pathway. *Brazilian Journal of Medical and Biological Research*.

[34] He S., Hou X., Xu X. (2015). Quantitative proteomic analysis reveals heat stress-induced injury in rat small intestine via activation of the MAPK and NF-*κ*B signaling pathways. *Molecular BioSystems*.

[35] Zhang T., Yin Y. C., Ji X. (2019). AT1R knockdown confers cardioprotection against sepsis-induced myocardial injury by inhibiting the MAPK signaling pathway in rats. *Journal of Cellular Biochemistry*.

[36] Tang Z., Yang C., Zuo B. (2019). Taxifolin protects rat against myocardial ischemia/reperfusion injury by modulating the mitochondrial apoptosis pathway. *PeerJ*.

[37] Suchal K., Malik S., Gamad N. (2016). Kaempferol attenuates myocardial ischemic injury via inhibition of MAPK signaling pathway in experimental model of myocardial ischemia-reperfusion injury. *Oxidative Medicine and Cellular Longevity*.

[38] Yuan R., Huang L., Du L. J. (2019). Dihydrotanshinone exhibits an anti-inflammatory effect in vitro and in vivo through blocking TLR4 dimerization. *Pharmacological Research*.

[39] Zhou D., Zhu Y., Ouyang M. Z. (2018). Knockout of Toll-like receptor 4 improves survival and cardiac function in a murine model of severe sepsis. *Molecular Medicine Reports*.

[40] Topkara V. K., Evans S., Zhang W. (2011). Therapeutic targeting of innate immunity in the failing heart. *Journal of Molecular and Cellular Cardiology*.

[41] Xu X., Rui S., Chen C. (2020). Protective effects of astragalus polysaccharide nanoparticles on septic cardiac dysfunction through inhibition of TLR4/NF-*κ*B signaling pathway. *International Journal of Biological Macromolecules*.

[42] Wei X., Meng X., Yuan Y., Shen F., Li C., Yang J. (2018). Quercetin exerts cardiovascular protective effects in LPS-induced dysfunction in vivo by regulating inflammatory cytokine expression, NF-*κ*B phosphorylation, and caspase activity. *Molecular and Cellular Biochemistry*.

[43] Chen J., Kieswich J. E., Chiazza F. (2016). IkappaB kinase inhibitor attenuates sepsis-induced cardiac dysfunction in CKD. *Journal of the American Society of Nephrology*.

[44] Hotchkiss R. S., Monneret G., Payen D. (2013). Immunosuppression in sepsis: a novel understanding of the disorder and a new therapeutic approach. *The Lancet Infectious Diseases*.

[45] Kumar A., Thota V., Dee L., Olson J., Uretz E., Parrillo J. E. (1996). Tumor necrosis factor alpha and interleukin 1beta are responsible for in vitro myocardial cell depression induced by human septic shock serum. *The Journal of Experimental Medicine*.

[46] Sun B., Xiao J., Sun X. B., Wu Y. (2013). Notoginsenoside R1 attenuates cardiac dysfunction in endotoxemic mice: an insight into oestrogen receptor activation and PI3K/Akt signalling. *British Journal of Pharmacology*.

[47] Kumar A., Kumar A., Paladugu B., Mensing J., Parrillo J. E. (2007). Transforming growth factor-beta1 blocks in vitro cardiac myocyte depression induced by tumor necrosis factor-alpha, interleukin-1beta, and human septic shock serum. *Critical Care Medicine*.

[48] Levine A. B., Punihaole D., Levine T. B. (2012). Characterization of the role of nitric oxide and its clinical applications. *Cardiology*.

[49] Pathan N., Franklin J. L., Eleftherohorinou H. (2011). Myocardial depressant effects of interleukin 6 in meningococcal sepsis are regulated by p38 mitogen-activated protein kinase. *Critical Care Medicine*.

[50] Hu J., Tang Z., Xu J. (2019). The inhibitor of interleukin-3 receptor protects against sepsis in a rat model of cecal ligation and puncture. *Molecular Immunology*.

[51] Tanaka T., Narazaki M., Kishimoto T. (2016). Immunotherapeutic implications of IL-6 blockade for cytokine storm. *Immunotherapy*.

[52] Wu C. L., Wu Q. Y., Du J. J. (2015). Calcium-sensing receptor in the T lymphocyte enhanced the apoptosis and cytokine secretion in sepsis. *Molecular Immunology*.

