# Supplementary figures and images for: Uncovering the Molecular Mechanism of the Qiang-Xin 1 Formula on Sepsis-Induced Cardiac Dysfunction Based on Systems Pharmacology
Source: Oxid Med Cell Longev. 2020 Aug 27;2020:3815185. doi: 10.1155/2020/3815185 (PMC7474398; doi:10.1155/2020/3815185)

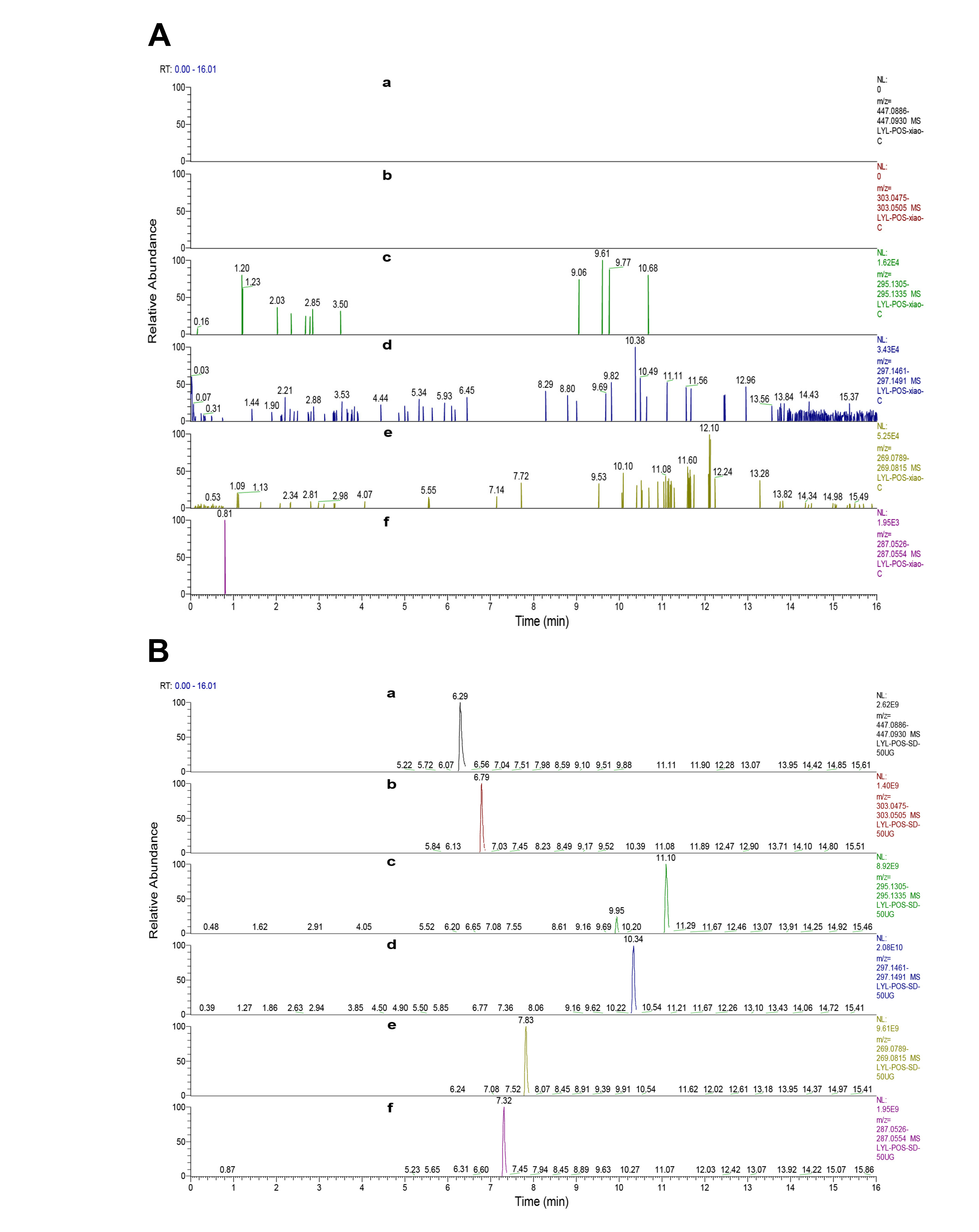

Supplement: Supplementary 1 — Figure S1: chromatograms of the six main bioactive compounds in rat plasma. (A) Blank plasma. (B) Blank rat plasma spiked with the six main bioactive compounds: (a) taxifolin, (b) quercetin, (c) tanshinone IIA, (d) cryptotanshinone, (e) formononetin, and (f) kaempferol. [file 3815185.f1.zip › Figure S1.tif]
