# Supplementary material for: Uncovering the Molecular Mechanism of the Qiang-Xin 1 Formula on Sepsis-Induced Cardiac Dysfunction Based on Systems Pharmacology
Source: Oxid Med Cell Longev. 2020 Aug 27;2020:3815185. doi: 10.1155/2020/3815185 (PMC7474398; doi:10.1155/2020/3815185)
Supplement: Supplementary 2 — Table S1: the potential targets for the 63 bioactive compounds in the QX1 formula. [file 3815185.f2.docx]

**Table S1. The potential targets for the 63 bioactive compounds in QX1 formula**

| **NO.** | **Compound** | **Target** |
| --- | --- | --- |
| 1 | palmitic acid | ADH1B、BCL2、COL1A1、F2、NOS2、NOS3、PPARG、PTGS1、PTGS2、TNF |
| 2 | quercetin | JUN、ACACA、AHR、ALOX5、AR、BCL2、COL1A1、COL3A1、CYP1A2、EGF、EGFR、ESR1、ESR2、F10、F2、F3、GJA1、GSK3β、GSTM1、GSTM2、GSTP1、HMOX1、HSPA5、IFNG、IL1B、IL2、IL6、INSR、KCNH2、MAPK1、MAPK14、MMP1、MMP2、MMP3、MPO、NOS2、NOS3、NQO1、PIK3CG、PLAT、PLAU、PON1、PPARG、PTGER3、PTGS1、PTGS2、SCN5A、SELE、SOD1、SULT1E1、THBD、TNF、TP53、VCAM1、VEGFA、XDH |
| 3 | Jaranol | AR、ESR1、ESR2、GSK3β、MAPK14、NOS2、PPARG、PTGS1、PTGS2、SCN5A |
| 4 | (2R)-2-[(3S,5R,10S,13R,14R,16R,17R)-3,16-dihydroxy-4,4,10,13,14-pentamethyl-2,3,5,6,12,15,16,17-octahydro-1H-cyclopenta[a]phenanthren-17-yl]-6-methylhept-5-enoic acid | AR、NR3C1、NR3C2 |
| 5 | trametenolic acid | AR、ESR1、NR3C1、NR3C2 |
| 6 | Cerevisterol | AR、ESR1、NR3C1、NR3C2 |
| 7 | hederagenin | ADH1B、ADRA1B、AR、CHRM2、ESR1、F2、GABRA6、NOS3、NR3C1、PDE3A、PPARG、PTGS1、PTGS2、SCN5A、SLC6A2 |
| 8 | n-coumaroyltyramine | ADRB2、EGFR、ESR1、GSK3β、LTA4H、MAPK14、PPARG、PTGS1 |
| 9 | isorhamnetin | AKR1B1、AR、ESR1、ESR2、F2、F7、GSK3β、MAPK14、NOS2、NOS3、PIK3CG、PPARG、PTGS1、PTGS2、XDH |
| 10 | beta-sitosterol | JUN、ADRA1A、ADRA1B、ADRB2、AR、BCL2、CHRM2、ESR1、ESR2、F2、GSK3β、HTR2A、KCNH2、MAPK14、NOS2、NR3C1、PDE3A、PIK3CG、PON1、PPARG、PTGS1、PTGS2、SCN5A、SLC6A4 |
| 11 | 3,9-di-O-methylnissolin | ADRA1B、ADRA2C、ADRB1、ADRB2、AR、ESR1、ESR2、F2、GSK3β、MAPK14、NOS2、NOS3、PDE3A、PPARG、PTGS1、PTGS2、SCN5A |
| 12 | Bifendate | AR、ESR1、F2、GSK3β、KCNMA1、KDR、NOS2、PTGS1、PTGS2 |
| 13 | formononetin | JUN、ADRA1A、ADRB2、AR、ESR1、ESR2、F2、GSK3β、MAPK14、NOS2、NOS3、PDE3A、PPARG、PTGS1、PTGS2、SLC6A4 |
| 14 | Calycosin | ADRB2、AR、ESR1、ESR2、GSK3β、MAPK14、NOS2、PDE3A、PPARG、PTGS1、PTGS2 |
| 15 | kaempferol | NOS2、INSR、ESR1、BCL2、ALOX5、PTGS2、 AKR1C3、TNF、ESR2、MMP1、JUN、SELE、VCAM1、XDH、MAPK8、CYP1A2、GSTP1、HMOX1、GSTM1、AHR、GSTM2、PTGS1、F2、AR、PPARG、NOS3、F7、SLC6A2、CHRM2、ADRA1B、MAPK14、GSK3β、PIK3CG |
| 16 | (2R)-5,7-dihydroxy-2-(4-hydroxyphenyl)chroman-4-one | AR、ESR1、ESR2、GSK3β、MAPK14、NOS2、NR3C1、NR3C2、PPARG、PTGS1、PTGS2 |
| 17 | Poriferasterol | AR、ESR1、NR3C1、NR3C2 |
| 18 | (-)-taxifolin | AR、ESR1、ESR2、GSK3β、MAPK14、NOS2、PIK3CG、PPARG、PTGS1、PTGS2 |
| 19 | Dehydrotanshinone II A | ADRA1A、ADRB2、AR、ESR1、ESR2、F2、GSK3β、HTR2A、MAPK14、NOS2、PPARG、PTGS2、SCN5A |
| 20 | Chryseriol | AR、ESR1、ESR2、GSK3β、MAPK14、NOS2、PIK3CG、PPARG、PTGS1、PTGS2 |
| 21 | taxifolin | AKR1B1、AR、ESR1、ESR2、GSK3β、MAPK14、NOS2、PIK3CG、PPARG、PTGS1、PTGS2 |
| 22 | eriodictyol | AR、ESR1、ESR2、GSK3β、HMOX1、MAPK14、NOS2、NQO1、PIK3CG、PPARG、PTGS1、PTGS2 |
| 23 | 2-isopropyl-8-methylphenanthrene-3,4-dione | ADRA1A、ADRA1B、ADRB2、AR、CHRM2、ESR1、ESR2、F2、GSK3β、HTR2A、MAPK14、NOS2、NOS3、PIK3CG、PPARG、PTGS1、PTGS2、SCN5A、SLC6A4 |
| 24 | 3α-hydroxytanshinoneⅡa | ADRB2、AR、ESR1、ESR2、F2、GSK3β、NOS2、PPARG、PTGS2、SCN5A |
| 25 | (E)-3-[2-(3,4-dihydroxyphenyl)-7-hydroxy-benzofuran-4-yl]acrylic acid | ESR1、ESR2、GSK3β、MAPK14、PPARG、PTGS2 |
| 26 | formyltanshinone | AR、ESR1、ESR2、F2、GSK3β、MAPK14、NOS2、PIK3CG、PPARG、PTGS2 |
| 27 | Przewaquinone B | AR、ESR1、ESR2、F2、GSK3β、MAPK14、NOS2、PIK3CG、PPARG、PTGS2 |
| 28 | przewaquinone c | ADRA1A、ADRB2、AR、CHRM2、ESR1、ESR2、F2、GSK3β、MAPK14、NOS2、NOS3、PIK3CG、PTGS1、PTGS2、SCN5A |
| 29 | przewaquinone f | AR、ESR1、ESR2、F2、GSK3β、NOS2、PPARG、PTGS2 |
| 30 | sclareol | AR、ESR1、F2、PTGS2 |
| 31 | tanshinaldehyde | ADRB2、AR、ESR1、ESR2、F2、GSK3β、HTR2A、NOS2、PPARG、PTGS2 |
| 32 | Tanshinol A | AR、ESR1、ESR2、F2、GSK3β、MAPK14、NOS2、PIK3CG、PPARG、PTGS2 |
| 33 | Danshenol B | AR、ESR1、ESR2、F2、NOS2、NR3C1、PTGS2 |
| 34 | Danshenol A | AR、ESR1、ESR2、F10、F2、GSK3β、KCNH2、KCNMA1、MAPK14、NOS2、PIK3CG、PPARG、PTGS1、PTGS2、SCN5A |
| 35 | cryptotanshinone | ADRA1A、ADRA1B、ADRB2、AR、CHRM2、ESR1、ESR2、F2、GSK3β、NOS2、PTGS1、PTGS2、SCN5A、TNF |
| 36 | danshenspiroketallactone | ADRA1A、ADRA1B、ADRB2、AR、CHRM2、ESR1、ESR2、F2、GSK3β、MAPK14、NOS2、NOS3、PTGS1、PTGS2、SCN5A、SLC6A4 |
| 37 | deoxyneocryptotanshinone | ADRA1A、ADRA1B、ADRB2、AR、CHRM2、ESR1、ESR2、F2、GSK3β、NOS2、NOS3、PTGS1、PTGS2、SCN5A |
| 38 | dihydrotanshinoneⅠ | ADRA1A、ADRA1B、ADRB2、AR、ESR1、ESR2、F2、GSK3β、MAPK14、NOS2、NOS3、PIK3CG、PPARG、PTGS1、PTGS2、SCN5A |
| 39 | isocryptotanshi-none | ADRA1A、ADRA1B、ADRB2、AR、CHRM2、ESR1、ESR2、F10、F2、GSK3β、NOS2、PTGS1、PTGS2、SCN5A |
| 40 | Isotanshinone II | ADRA1A、ADRB2、AR、CHRM2、ESR1、ESR2、F2、GSK3β、NOS2、PTGS2、SCN5A |
| 41 | Isotanshinone I | ADRB2、AR、ESR1、ESR2、F2、GSK3β、MAPK14、NOS2、PIK3CG、PPARG、PTGS1、PTGS2 |
| 42 | manool | AR、ESR1 |
| 43 | methyltanshinonate | ADRB2、AR、ESR1、ESR2、F2、GSK3β、HTR2A、NOS2、PPARG、PTGS2、SCN5A |
| 44 | miltionone Ⅰ | ADRA1A、ADRA1B、ADRB2、AR、CHRM2、ESR1、ESR2、F10、F2、GSK3β、HTR2A、NOS2、NR3C1、PTGS1、PTGS2、SCN5A |
| 45 | Miltirone | ADRA1A、ADRA1B、ADRA2C、ADRB2、AR、CHRM2、ESR1、ESR2、F2、GSK3β、NOS2、NOS3、PTGS1、PTGS2、SCN5A |
| 46 | neocryptotanshinone | ADRA1B、ADRB2、AR、ESR1、ESR2、F2、GSK3β、NOS2、PPARG、PTGS1、PTGS2、SCN5A |
| 47 | prolithospermic acid | AR、ESR1、ESR2、F2、GSK3β、MAPK14、NOS2、PPARG、PTGS1、PTGS2 |
| 48 | Tanshindiol B | AR、ESR1、ESR2、F2、GSK3β、NOS2、PTGS2 |
| 49 | Przewaquinone E | AR、ESR1、ESR2、F2、GSK3β、NOS2、PTGS2 |
| 50 | tanshinone iia | JUN、ADRA1A、ADRB2、AR、BCL2、CHRM2、CYP1A2、ECE1、ESR1、ESR2、F2、FASN、GSK3β、MAPK14、NOS2、NOS3、PTGS2、SCN5A、TP53 |
| 51 | tanshinone Ⅵ | AR、ESR1、ESR2、F10、F2、GSK3β、MAPK14、NOS2、PPARG、PTGS1、PTGS2、SCN5A |
| 52 | 2-(4-hydroxyphenyl)ethyl (E)-3-(4-hydroxyphenyl)prop-2-enoate | ADRB2、ESR1、LTA4H、MAPK14、PPARG |
| 53 | Schisanhenol | AR、ESR1、ESR2、F2、NOS2、PPARG、PTGS2、SCN5A |
| 54 | 4,7-dimethyl-7-(4-methylpent-3-enyl)bicyclo[2.2.1]heptan-3-ol | SLC6A2 |
| 55 | Angeloylgomisin H | ADRA1B、CHRM2、F2 |
| 56 | Schizandrer B | ESR1、PTGS2 |
| 57 | Clupanodonic acid | NOS2、PTGS1、PTGS2 |
| 58 | Gomisin D | ESR1、PTGS2 |
| 59 | Gomisin H | ESR1、PTGS2 |
| 60 | Schisanhenol acetate | AR、ESR1、KCNMA1、PTGS2 |
| 61 | Schizonepetoside A | AR、ESR1、KCNMA1、PTGS2 |
| 62 | Thujyalcohol | F2、NOS2、NOS3、PTGS2、SLC6A2 |
| 63 | kaempferol-3-O-α-L-rhamnoside | PTGS2 |
