# Supplementary material for: Uncovering the Molecular Mechanism of the Qiang-Xin 1 Formula on Sepsis-Induced Cardiac Dysfunction Based on Systems Pharmacology
Source: Oxid Med Cell Longev. 2020 Aug 27;2020:3815185. doi: 10.1155/2020/3815185 (PMC7474398; doi:10.1155/2020/3815185)
Supplement: Supplementary 3 — Table S2: the degree of protein targets corresponding to potential compounds in the QX1 formula. [file 3815185.f3.docx]

**Table S2 The degree of protein targets corresponding to potential compounds in QX1 formula**

| **NO.** | **Target** | **Degree** | **species** |
| --- | --- | --- | --- |
| 1 | ESR1 | 57 | human |
| 2 | PTGS2 | 55 | human |
| 3 | AR | 52 | human |
| 4 | NOS2 | 46 | human |
| 5 | ESR2 | 43 | human |
| 6 | GSK3β | 43 | human |
| 7 | F2 | 40 | human |
| 8 | PPARG | 35 | human |
| 9 | PTGS1 | 32 | human |
| 10 | MAPK14 | 30 | human |
| 11 | ADRB2 | 23 | human |
| 12 | SCN5A | 23 | human |
| 13 | PIK3CG | 16 | human |
| 14 | NOS3 | 15 | human |
| 15 | ADRA1A | 14 | human |
| 16 | ADRA1B | 14 | human |
| 17 | CHRM2 | 14 | human |
| 18 | NR3C1 | 9 | human |
| 19 | HTR2A | 6 | human |
| 20 | BCL2 | 5 | human |
| 21 | F10 | 5 | human |
| 22 | JUN | 1 | human |
| 23 | NR3C2 | 5 | human |
| 24 | PDE3A | 5 | human |
| 25 | KCNMA1 | 4 | human |
| 26 | SLC6A2 | 4 | human |
| 27 | SLC6A4 | 4 | human |
| 28 | TNF | 4 | human |
| 29 | CYP1A2 | 3 | human |
| 30 | HMOX1 | 3 | human |
| 31 | KCNH2 | 3 | human |
| 32 | XDH | 3 | human |
| 33 | ADH1B | 2 | human |
| 34 | ADRA2C | 2 | human |
| 35 | AHR | 2 | human |
| 36 | AKR1B1 | 2 | human |
| 37 | ALOX5 | 2 | human |
| 38 | COL1A1 | 2 | human |
| 39 | EGFR | 2 | human |
| 40 | F7 | 2 | human |
| 41 | GSTM1 | 2 | human |
| 42 | GSTM2 | 2 | human |
| 43 | GSTP1 | 2 | human |
| 44 | INSR | 2 | human |
| 45 | LTA4H | 2 | human |
| 46 | MMP1 | 2 | human |
| 47 | NQO1 | 2 | human |
| 48 | PON1 | 2 | human |
| 49 | SELE | 2 | human |
| 50 | TP53 | 2 | human |
| 51 | VCAM1 | 2 | human |
| 52 | ACACA | 1 | human |
| 53 | ADRB1 | 1 | human |
| 54 | AKR1C3 | 1 | human |
| 55 | COL3A1 | 1 | human |
| 56 | ECE1 | 1 | human |
| 57 | EGF | 1 | human |
| 58 | F3 | 1 | human |
| 59 | FASN | 1 | human |
| 60 | GABRA6 | 1 | human |
| 61 | GJA1 | 1 | human |
| 62 | HSPA5 | 1 | human |
| 63 | IFNG | 1 | human |
| 64 | IL1B | 1 | human |
| 65 | IL2 | 1 | human |
| 66 | IL6 | 1 | human |
| 67 | KDR | 1 | human |
| 68 | MAPK1 | 1 | human |
| 69 | MAPK8 | 1 | human |
| 70 | MMP2 | 1 | human |
| 71 | MMP3 | 1 | human |
| 72 | MPO | 1 | human |
| 73 | PLAT | 1 | human |
| 74 | PLAU | 1 | human |
| 75 | PTGER3 | 1 | human |
| 76 | SOD1 | 1 | human |
| 77 | SULT1E1 | 1 | human |
| 78 | THBD | 1 | human |
| 79 | VEGFA | 1 | human |
