# Supplementary material for: Uncovering the Molecular Mechanism of the Qiang-Xin 1 Formula on Sepsis-Induced Cardiac Dysfunction Based on Systems Pharmacology
Source: Oxid Med Cell Longev. 2020 Aug 27;2020:3815185. doi: 10.1155/2020/3815185 (PMC7474398; doi:10.1155/2020/3815185)
Supplement: Supplementary 4 — Table S3: the information of the target-pathway network in the QX1 formula. [file 3815185.f4.docx]

**Table S3 The information of target-pathways network in QX1 formula**

| **Pathways** | **Targets** | **Degree** |
| --- | --- | --- |
| HIF-1 signaling pathway | PIK3CG, EGFR, MAPK1, IL6, BCL2, VEGFA, IFNG, NOS3, NOS2, EGF, INSR | 11 |
| PI3K-Akt signaling pathway | PIK3CG, EGFR, IL6, COL3A1, TP53, KDR, MAPK1, CHRM2, BCL2, GSK3β, VEGFA,NOS3, COL1A1, EGF, INSR, IL2 | 16 |
| cGMP-PKG signaling pathway | PIK3CG, KCNMA1, MAPK1, ADRB2, ADRB1, ADRA1B, ADRA1A, PDE3A, NOS3,ADRA2C, INSR | 11 |
| Regulation of lipolysis in adipocytes | PIK3CG, ADRB2, PTGER3, ADRB1, PTGS2, PTGS1, INSR | 7 |
| VEGF signaling pathway | PIK3CG, MAPK1, PTGS2, MAPK14, VEGFA, NOS3, KDR | 7 |
| Complement and coagulation cascades | PLAT, THBD, F10, F3, F2, F7, PLAU | 7 |
| T cell receptor signaling pathway | PIK3CG, MAPK1, TNF, MAPK14, JUN, GSK3β, IFNG, IL2 | 8 |
| Calcium signaling pathway | EGFR, ADRB2, PTGER3, ADRB1, CHRM2, ADRA1B, ADRA1A, NOS3, NOS2, HTR2A | 10 |
| Toll-like receptor signaling pathway | PIK3CG, MAPK1, IL6, TNF, MAPK14, JUN, IL1B, MAPK8 | 8 |
| Adrenergic signaling in cardiomyocytes | PIK3CG, MAPK1, ADRB2, ADRB1, MAPK14, BCL2, ADRA1B, ADRA1A, SCN5A | 9 |
| Neurotrophin signaling pathway | PIK3CG, MAPK1, MAPK14, JUN, GSK3β, BCL2, TP53, MAPK8 | 8 |
| NOD-like receptor signaling pathway | MAPK1, IL6, TNF, MAPK14, IL1B, MAPK8 | 6 |
| ErbB signaling pathway | PIK3CG, EGFR, MAPK1, JUN, GSK3β, MAPK8, EGF | 7 |
| Type II diabetes mellitus | PIK3CG, MAPK1, TNF, MAPK8, INSR | 5 |
| NF-kappa B signaling pathway | VCAM1, TNF, PTGS2, BCL2, IL1B, PLAU | 6 |
| Platelet activation | PIK3CG, MAPK1, MAPK14, COL3A1, PTGS1, NOS3, COL1A1 | 7 |
| GnRH signaling pathway | EGFR, MAPK1, MAPK14, JUN, MAPK8, MMP2 | 6 |
| Cytokine-cytokine receptor interaction | EGFR, IL6, TNF, VEGFA, IFNG, IL1B, EGF, KDR, IL2 | 9 |
| Insulin signaling pathway | PIK3CG, MAPK1, GSK3β, ACACA, FASN, MAPK8, INSR | 7 |
| Arachidonic acid metabolism | AKR1C3, PTGS2, PTGS1, LTA4H, ALOX5 | 5 |
| Choline metabolism in cancer | PIK3CG, EGFR, MAPK1, JUN, MAPK8, EGF | 6 |
| MAPK signaling pathway | EGFR, MAPK1, TNF, MAPK14, JUN, TP53, IL1B, MAPK8, EGF | 9 |
| Fc epsilon RI signaling pathway | PIK3CG, MAPK1, TNF, MAPK14, MAPK8 | 5 |
| Drug metabolism - cytochrome P450 | GSTM1, GSTM2, ADH1B, CYP1A2, GSTP1 | 5 |
| Serotonergic synapse | MAPK1, PTGS2, SLC6A4, PTGS1, ALOX5, HTR2A | 6 |
| Metabolism of xenobiotics by cytochrome P450 | GSTM1, GSTM2, ADH1B, CYP1A2, GSTP1 | 5 |
| AMPK signaling pathway | PIK3CG, PPARG, ACACA, FASN, ADRA1A, INSR | 6 |
| Type I diabetes mellitus | TNF, IFNG, IL1B, IL2 | 4 |
| Ras signaling pathway | PIK3CG, EGFR, MAPK1, VEGFA, MAPK8, EGF, INSR, KDR | 8 |
| B cell receptor signaling pathway | PIK3CG, MAPK1, JUN, GSK3β | 4 |
